# Quartet Fiduccia–Mattheyses revisited for larger phylogenetic studies

**DOI:** 10.1093/bioinformatics/btad332

**Published:** 2023-06-07

**Authors:** Sharmin Akter Mim, Md Zarif-Ul-Alam, Rezwana Reaz, Md Shamsuzzoha Bayzid, Mohammad Saifur Rahman

**Affiliations:** Department of Computer Science and Engineering, Bangladesh University of Engineering and Technology, Dhaka 1205, Bangladesh; Department of Computer Science and Engineering, Bangladesh University of Engineering and Technology, Dhaka 1205, Bangladesh; Department of Computer Science and Engineering, Bangladesh University of Engineering and Technology, Dhaka 1205, Bangladesh; Department of Computer Science and Engineering, Bangladesh University of Engineering and Technology, Dhaka 1205, Bangladesh; Department of Computer Science and Engineering, Bangladesh University of Engineering and Technology, Dhaka 1205, Bangladesh

## Abstract

**Motivation:**

With the recent breakthroughs in sequencing technology, phylogeny estimation at a larger scale has become a huge opportunity. For accurate estimation of large-scale phylogeny, substantial endeavor is being devoted in introducing new algorithms or upgrading current approaches. In this work, we endeavor to improve the Quartet Fiduccia and Mattheyses (QFM) algorithm to resolve phylogenetic trees of better quality with better running time. QFM was already being appreciated by researchers for its good tree quality, but fell short in larger phylogenomic studies due to its excessively slow running time.

**Results:**

We have re-designed QFM so that it can amalgamate millions of quartets over thousands of taxa into a species tree with a great level of accuracy within a short amount of time. Named “QFM Fast and Improved (QFM-FI)”, our version is 20 000× faster than the previous version and 400× faster than the widely used variant of QFM implemented in PAUP* on larger datasets. We have also provided a theoretical analysis of the running time and memory requirements of QFM-FI. We have conducted a comparative study of QFM-FI with other state-of-the-art phylogeny reconstruction methods, such as QFM, QMC, wQMC, wQFM, and ASTRAL, on simulated as well as real biological datasets. Our results show that QFM-FI improves on the running time and tree quality of QFM and produces trees that are comparable with state-of-the-art methods.

**Availability and implementation:**

QFM-FI is open source and available at https://github.com/sharmin-mim/qfm_java.

## 1 Introduction

The notion of evolution is central to biology, with significant applications in a range of domains, such as molecular biology, virology, ecology, physiology, cancer genomics, and epidemiology (Linder and Warnow 2005, Shi and Wang 2011, Liu *et al.* 2013, Schwartz and Schäffer 2017, MacLean *et al.* 2020). Because of genomic variability induced by events like Incomplete Lineage Sorting (ILS), Gene Duplication and Loss, Horizontal Gene Transfer, and hybridization events, reconstruction of species phylogeny (evolutionary linkages among species) from genes sampled throughout the genome becomes incredibly difficult (Maddison 1997).

Standard approaches for estimating species trees, such as concatenation, can be statistically inconsistent in the presence of gene tree heterogeneity (Roch and Steel 2015). Therefore, “summary methods” are becoming increasingly popular due to their high accuracy and statistical guarantee under ILS (Bayzid and Warnow 2013).

Quartet-based summary methods have gained substantial interest as quartets (4-leaf unrooted gene trees) do not contain the “anomaly zone” (Degnan and Rosenberg 2006, 2009, Degnan 2013), a condition where the most probable gene tree topology may not be identical to the species tree topology. ASTRAL (Mirarab *et al.* 2014b, Mirarab and Warnow 2015, Zhang *et al.* 2018), one of the most accurate and widely used species tree estimation methods, relies on dividing gene trees into quartets, a feature that allows it to address ILS and may contribute to its high accuracy. A recent method called ASTRAL-Pro (Zhang *et al.* 2020) aims to calculate a single-copy tree (the species tree) to maximize the overall similarity to the input gene trees by applying dynamic programing. ASTRAL-Pro 2 (Zhang and Mirarab 2022) adopts a placement-based optimization algorithm for significantly better scalability without sacrificing accuracy.

Another class of quartet-based methods is “quartet amalgamation techniques.” The broader impact and notable advantage of quartet amalgamation techniques over ASTRAL is that they can be used outside the context of gene tree estimation. A recent study, QT-GILD (Mahbub *et al.* 2022), showed that quartet distribution inferred from incomplete gene trees can be imputed (using techniques from machine learning), and amalgamating the imputed quartets may result in substantially higher accuracy in species tree estimation compared to ASTRAL in the presence of missing data.

Using semidefinite programing, Quartet Max-Cut (QMC) seeks to obtain a phylogenetic tree, which is congruent with the highest number of input quartets (Snir and Rao 2012). Quartets with the appropriate weighting can boost the performance of quartet-based approaches significantly (Ranwez and Gascuel 2001, Holland *et al.* 2013). QMC is extended in order to support weighted quartet trees, which is termed as wQMC (Avni *et al.* 2015).

Reaz *et al.* (2014) proposed a new quartet-based method, Quartet Fiduccia–Mattheyses (QFM), and showed that QFM outperforms QMC in terms of tree quality. QFM is being widely used in important phylogenetic studies (Mason *et al.* 2016, Moumi *et al.* 2019, Zhou *et al.* 2022), especially along with SVDquartets method (Chifman and Kubatko 2014). Rahman (2018) conducted an experiment where QFM was compared against ASTRAL and showed that Disk-covering Method (Roshan *et al.* 2004) boosted QFM outperforms ASTRAL on the 37-taxa simulated dataset. But excessively high running time of QFM prevented the author to conduct the experiment on larger datasets. A variant of QFM is implemented in PAUP* (Swofford 2003) (referred as “QFM-PAUP” subsequently in this article) to construct phylogenetic tree by amalgamating unweighted quartets generated from SVDQuartets method (Chifman and Kubatko 2014). While this variant is faster, our experiments suggest that it is still not suitable for large-scale phylogeny estimation. Very recently, Mahbub *et al.* (2021) gave a weighted formulation of QFM (wQFM), which can amalgamate weighted quartets. Even in that study, the largest dataset they experimented with had only 100 taxa.

Meanwhile, the demand for phylogeny estimation methods that can be quite accurate on ultra-large datasets is increasing as more sequence datasets become obtainable because of the advancement of next-generation sequencing technologies. NJMerge, a method for inferring phylogeny in a massive scale, does not ensure that it will output a species tree as it becomes collapsed in some cases at the time of searching and establishing legal sibling-hood (Molloy and Warnow 2018, 2019). Le *et al.* (2021) explored different variants of constrained-INC (Zhang *et al.* 2019) and found unsatisfactory gene tree accuracy and optimistic species tree quality when compared to conventional phylogenetic tree building approaches, despite the fact that just one model condition in their experiment included more than 1000 sequences. Han and Molloy (2023) have recently developed TREE-QMC, which is based on wQMC and offers a fast method for constructing the quartet graph directly from input gene trees without the need for explicitly computing the weighted quartet distributions as in the case of wQMC and wQFM.

The contributions of this article are summarized as follows:

- We have modified the QFM algorithm to make it scale better with increased number of taxa and quartets. We have significantly improved the running time complexity of the algorithm through the use of suitable data structures.
- We have also made modifications to QFM algorithm that resulted in improvement in the tree quality.
- We have demonstrated the performance of the modified QFM algorithm through an extensive experimental study. Our experimental design includes several simulated as well as biological datasets in which QFM of Reaz *et al.* (2014) does not scale to.

The rest of the article is organized as follows. In Section 2, we briefly describe datasets and explain the improvements made to the QFM algorithm, followed by its complexity analysis. In Section 3, we describe the extensive experimental studies we have conducted and showcase the results. This is followed by a brief discussion subsection. Finally, Section 4 concludes the article.

## 2 Materials and methods

### 2.1 Datasets

#### 2.1.1 Simulated datasets

**Figure 1.**
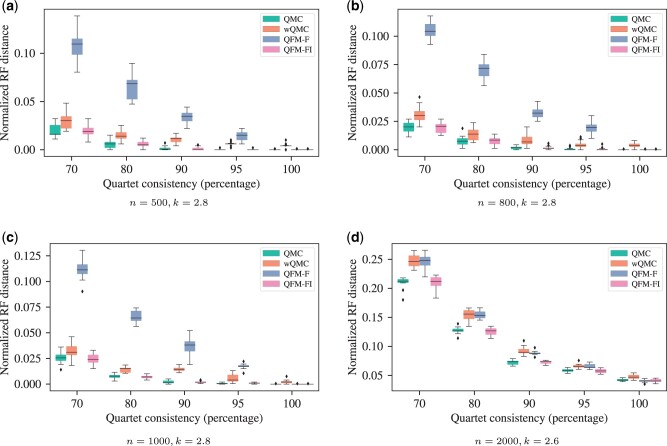
Box plot of nRF distance of trees produced by QMC, wQMC, QFM-F, and QFM-FI for various quartet consistency levels and number of taxa (*n*). For *n *=* *500, 800, and 1000, *k* has been set to 2.8. For *n *=* *2000, we have used *k *=* *2.6. (a) n=500,k=2.8; (b) n=800,k=2.8; (c) n=1000,k=2.8; (d) n=2000,k=2.6.

- **Simulated dataset-1.** Following the approach in Snir and Rao (2012), Reaz *et al.* (2014), and Avni *et al.* (2015), we have generated model species trees for varied number of taxa (species), denoted as *n*, using the r8s software (Sanderson 2003). Specifically, we have used n∈{25,50,100,200,300,400,500,800,1000,2000,3000}. Then a set of noisy and noiseless model conditions have been formulated by varying the consistency level (*c*) parameter. Here, *c* indicates the percentage of quartets that agree with the model species tree topology. We have used c∈{70%,80%,90%,95%, 100%}. The total number of quartets (*m*) in a dataset has been determined by another parameter *k*, such that $m=n^{k}$. We have used k∈{1.5,2,2.8} for n≤800. For larger values of *n*, we have used k∈{1.5,2}. Two sets of quartets (one weighted and another one unweighted) have been extracted from each model species tree for the different combinations of *c* and *k* by utilizing the software developed and employed in (Avni *et al.* 2015) Thus, this dataset consists of 150 different model conditions and among them 120 model conditions are noisy (i.e. c<100%) and 30 model conditions are noiseless (i.e. c=100%).
  Additionally, we have used two other settings of (n,k)=(1000,2.8) (Fig. 1c) and (n,k)=(2000,2.6) (Fig. 1d) for the different consistency levels.
- **SATe dataset.** To compare our method with the QFM-PAUP, we have utilized the simulated nucleotide dataset used in Liu *et al.* (2009) as multiple sequence alignment (MSA) is required as input of SVDquartets method in PAUP*. We have used 22 out of 37 model conditions where number of taxa is either 100 or 500 and gap length is long, medium or short.
- **37-taxon simulated dataset.** This dataset, studied in Song *et al.* (2012), contains gene trees representing various model conditions with varied number of genes from 25 to 800, sequence length from 250 bp to its true length and ILS level as low (2×), moderate (1×), and high (0.5×).
- **100-taxon simulated dataset.** This dataset was experimented with in Mirarab and Warnow (2015). In each replicate there are 1000 true gene trees and number of taxa is 100 (plus 1 outgroup taxa).

We have used 20 replicates for each model conditions of all the simulated datasets except the 100-taxon simulated dataset where only 10 replicates are used.

Algorithm 1 Function for MFM_Bipartition

1: **Function** MFM_Bipartition($Q,P_{l},P_{r}$)

2:  **while** True **do**

3:   Set status of each taxon to FREE

4:   **for**q∈Q**do**

5:    Check status of *q* with respect to *P_l_*, *P_r_*.

6:    Calculate satisfied/violated/deferred score.

7:    **for** each taxa *t* of *q* **do**

8:     Check quartet status if *t* were to move to its opposite partition.

9:    **end for**

10:   **end for**

11:   Calculate initial partition score

12:   **while** There is a free taxon **do**

13:    **for** Each free taxon *t* in any partition **do**

14:     Calculate Gain as the increase in partition score if *t* switches partitions

15:    **end for**

16:    $t_{m}\leftarrow$ taxa with Maximum Gain

17:    Update (*P_l_*, *P_r_*) to reflect partition switching of *t_m_*

18:    $\mathrm{Status}(t_{m})\leftarrow \mathrm{LOCKED}$

19:    Log in a table (*t_m_*, Maximum Gain)

20:    **for** q ∈ set of relevant quartets of *t_m_* **do**

21:     **for** each free taxa in *q* **do**

22:      Subtract satisfied/violated/deferred score due to *t_m_*

23:      Add new satisfied/violated/deferred score due to *t_m_* moving to its opposite partition

24:     **end for**

25:    **end for**

26:   **end while**

27:   c← index of Log Table such that $\sum_{1}^{c}\mathrm{Maximum}\;\mathrm{Gain}$ is maximum. Call it MCGain

28:   **if** MCGain > 0 **then**

29:    Update (*P_l_*, *P_r_*) by rolling back to the switching of taxa that occurred in index *c* of Log Table.

30:   **else**

31:    Break

32:   **end if**

33:  **end while**

34:  **return** (*P_l_*, *P_r_*)

35: **end Function**

#### 2.1.2 Biological datasets

- **Plant dataset.** This dataset, taken from Wickett *et al.* (2014), includes 852 nuclear genes and 1 701 170 aligned sites from 103 taxa. There are 424 gene trees, generated from first and second codon position alignments after discarding genes with <50% taxon occupancy and gene fragments lacking more than 66% of their sites. Concatenated alignment of first and second codon positions, after exclusion of gene alignments with <50% taxon occupancy and gene fragments with <66% of their sites, was also available in this dataset.
- **Amniota dataset.** The amniota dataset (amino acid gene trees) is taken from Chiari *et al.* (2012), which consists 248 genes from 16 amniota taxa.
- **Avian dataset.** The avian dataset, which comprises 14 446 loci across 48 taxa, is taken from Jarvis *et al.* (2014).
- **Angiosperm dataset.** This dataset included 310 gene samples from 42 angiosperms and four outgroups, which is taken from Xi *et al.* (2014).

Quartets have been generated from the gene trees in 37-taxon simulated dataset, 100-taxon simulated dataset, and all biological datasets using the method described in Brodal *et al.* (2013). For the weighted setting (for wQMC and wQFM), the frequency of the quartets has been used as their respective weights.

### 2.2 QFM improvements

The QFM algorithm and the sub-routines it invokes are given in Supplementary Algorithms S1–S3 and Algorithm 1. We have not only significantly improved the running time complexity of QFM, but also improved the quality of the tree produced. When applying the modifications to the algorithm, we at first implemented a version which is much faster than QFM, but produces identical trees. We call this version “QFM Fast” or “QFM-F” in short. Subsequently, we tweaked the algorithm further so as to improve the tree quality as well. This version is referred to as “QFM Fast and Improved” or “QFM-FI” in short.

In our modified versions of QFM (QFM-F and QFM-FI), we have taken advantage of several efficient data structures as well as clever bookkeeping of certain information, which was not done in (Reaz *et al.* 2014). Let, *n* and *m* be the cardinality of taxa set *P* and quartet set *Q*, respectively. Now, we will go over the details of fast implementation of QFM algorithm.

- **Frequency counting.** At the time of storing the input quartets, we need to identify the quartets, which has same topology and count the occurrences of the same topological structure. This count is known as frequency of a quartet. We have applied hashing to make this identification and counting process much faster. While QFM, as described in Reaz *et al.* (2014) takes $O(m^{2})$ time for frequency counting of all quartets, our modified version only requires O(m) time. During this phase, the set of all distinct taxa is also extracted from the set of quartets. For each taxa, we maintain several pieces of information, such as, the partition the taxa belongs to, the quartets that have this taxa as a leaf node etc. These data are stored in another hash table, indexed by the taxon identifier.
- **Initial bi-partitioning.** Initial bi-partitioning consists of sorting *Q* in descending order of quartet frequency which requires O(mlogm) time and bi-partitioning *P* into left partition (*P_l_*) and right partition (*P_r_*), by examining each quartet in descending order of frequency and distributing its taxa in the two partitions following the rules described in Supplementary Algorithm S3. Since hashing is used to store the set of taxa, the checking and insertion of taxa of each quartet into a partition take O(1) time. So, the requisite time for *m* quartets is O(m). As a result, the worst case time complexity of initial bi-partitioning becomes O(mlogm)+O(m)∼O(mlogm) whereas in Reaz *et al.* (2014), it was $O(m^{2})+O(mn)∼O(m^{2})$.
- **MFM bi-partitioning.** In this step, the initial partition is perturbed to produce a modified partitioning with an improved partition score (see Algorithm 1). The major parts of the algorithm are as follows.

*Quartet status checking (Lines 4–10).* For each q∈Q, it is checked whether the quartet is satisfied, violated or deferred with respect to the initial partition and the corresponding numbers are updated. A quartet ((a,b),(c,d)) is satisfied with respect to *P_l_*, *P_r_*, if taxa *a* and *b* are in $P_{l}(P_{r})$ and *c* and *d* are in $P_{r}(P_{l})$. The quartet is violated if *a* and *c* are in one partition while *b* and *d* are in another. Alternately, it can be violated if *a* and *d* are in one partition and *b* and *c* are in another. A quartet is called deferred if at least three taxa are in same partition. The quartet status is further checked by simulating switching of partition for each of its four taxa separately. As the hash table of taxa set contains the information on which partition a taxa is in, the aforementioned status check takes O(1) time for one quartet. The required time for *m* quartets is thus O(m).

*Initial partitioning score (Line 11).* Initial partitioning score is calculated by subtracting initial violated score from initial satisfied score. This part needs O(1) time.

*Locking a free taxa (Lines 13–25).* We first measure the gain of each free taxa (i.e. increase in partition score if the taxon would be moved to its opposite partition), then taxon with the maximum gain is selected and locked. Since status checking is already done in previous step, it needs only O(1) time to measure gain for each taxon and O(n) for all *n* taxa. So, here the required time is ∼O(n).

*Locking all free taxa.* Since initially there are *n* free taxa, total required time for locking all free taxa is $O(n^{2})$. When a free taxon is locked, it is also moved to its opposite partition. So, status of all the quartets which contain this taxon (i.e. the relevant quartets) may also be changed. A quartet is a relevant quartet for all of its four taxa. So during the course of locking all free taxa, each quartet’s status gets checked four times, incurring O(m) time in total. As a result, locking all *n* taxa needs total $O(n^{2})+O(m)$ time.

*Calculation of maximum cumulative gain (Line 27).* It is done through a linear pass over the “log table,” thus requiring O(n) time.

Thus running time for one iteration of the outer loop of this routine (Lnes 2–33) is $O(n^{2})+O(m)+O(n)∼O(\max(n^{2},m))$ whereas it was $O(n^{3}m)$ in Reaz *et al.* (2014). Iteration continues until the value of maximum cumulative gain becomes ≤0. Therefore, the partition score after each iteration must increase by at least 1. Since partition score is the difference between the number of satisfied (*s*) and violated (*v*) quartets (see Reaz *et al.* 2014), it is an integer and its maximum value can be *m*. Therefore, it follows that the outer loop iterates no more than *m* times. Therefore, the running time of Algorithm 1 is $O(\max(mn^{2},m^{2}))$. Letting $k={\;\text{log}\;}_{n}m$, it becomes $O(\max(n^{k+2},n^{2k}))$ or $O(n^{\max(2k,k+2)})$.

- **Short quartet puzzle.** This step is same as in Snir *et al.* (2008) and Snir and Rao (2010) except that we have added a counting section, which counts the frequency of left quartet set *Q_l_* and right quartet set *Q_r_* before the next recursive call of Modified_SQP function (see Supplementary Algorithm S2). The function is recursively called on both pairs (*Q_l_*, *P_l_*) and (*Q_r_*, *P_r_*). Recursion terminates when there are only three taxa, at which point a depth one tree (i.e. a star of three taxa) is returned. After trees are returned from both recursive calls, they are merged using the dummy taxon [see Reaz *et al.* (2014) for details]. The running time of one call of Modified_SQP (without the recursive calls) is dictated by the time taken by the MFM bi-partitioning step.

Reaz *et al.* (2014) showed that the running time for one iteration of the outer loop of their implementation of MFM_Bipartition is $O(n^{3}m)=O(n^{k+3})$. And they did not provide an upper bound on the number of iterations. Based on the upper bound, we have provided above, their time complexity becomes, $O(n^{k+3}m)=O(n^{2k+3})$, while in our improved version, it is $O(n^{\max(2k,k+2)})$. We have thus considerably improved the running time of MFM_Bipartition.

We also modified the algorithm to improve the tree quality. When the violated quartets are modified with dummy taxon (Lines 11 and 12 of Supplementary Algorithm S2), several quartets may end up in the same topological structure. Let ((a,b),(c,d)) and ((a,b),(c,e)) be two quartets in *Q* in a call to Modified_SQP. Let *a*, *b* and *c* belonged to one partition and *d* and *e* in the other. In this scenario, *c* and *d* are called “deserted taxon” of these two quartets, respectively. Then both quartets will be modified so as to become $\left((a,b),(c,t_{d})\right)$ where *t_d_* is the dummy taxon. In such case, we have maintained only one copy of the quartets with identical structure and combined their frequencies. All the deferred quartets are sorted again according to frequency in each divide step, whereas sorting was done only in the first divide step in QFM of Reaz *et al.* (2014). With this modification, we have seen significant improvement in tree quality, as shown later in the article.

### 2.3 Complexity analysis

As before, we denote the number of taxa by *n*, the number of input quartets by *m* and we let $k={\;\text{log}\;}_{n}m$. As discussed in the previous section, one call of Modified_SQP, without the recursive calls, takes $O(n^{\max(2k,k+2)})$ time. Let the *n* taxa are split into *i* and *n–i* taxa in left (*P_l_*) and right (*P_r_*) partitions, respectively. The dummy taxon is added to both partitions. Let, *T*(*n*) denotes the total running time of QFM-FI algorithm for *n* taxa. Then


(1) $$\begin{array}{c} T(n)=T(i+1)+T(n-i+1)+O(n^{\max(2k,k+2)})\\ =T(i+1)+T(n-i+1)+cn^{\max(2k,k+2)} \end{array}.$$

The worst case ($T_{\mathrm{worst}}(n)$) occurs when partitioning in each recursive step is extremely skewed, i.e. one partition contains three taxa and the other contains the rest. Letting z=max(2k,k+2),


$$\begin{array}{cc} T_{\mathrm{worst}}(n) & =T(3)+T_{\mathrm{worst}}(n-1)+cn^{z}\\ & =T(3)+T(3)+T_{\mathrm{worst}}(n-2)+c{\left(n-1\right)}^{z}+cn^{z}\\ & =[T(3)+\cdots +T(3)]+T_{\mathrm{worst}}(n-(n-3))+[c4^{z}\\ & +c5^{z}+\cdots +c{\left(n-1\right)}^{z}+cn^{z}]\\ & =(n-2)T(3)+c\sum_{i=4}^{n}i^{z}\\ & \leq O(n)+cn^{z+1}=O(n^{z+1})=O(n^{\max(2k,k+2)+1})\\ & =O(\max(nm^{2},n^{3}m)) \end{array}.$$

We have also analyzed overall space complexity of QFM-FI, which is O(m+n) (see Supplementary Material for details).

## 3 Results and discussion

### 3.1 Comparison of tree quality on the basis of nRF

Comparison is performed in terms of normalized Robinson Foulds Distance (nRF) (see Supplementary Material for the definition of nRF).

#### 3.1.1 Comparison among QMC, wQMC, QFM-F, and QFM-FI

Using “simulated dataset-1,” we have conducted extensive experiments to compare QMC, wQMC, QFM-F, and QFM-FI, as demonstrated in Fig. 1 as well as Supplementary Tables S1 and S2. Like QFM-F and QFM-FI, the QMC algorithm takes unweighted quartets as input while wQMC expects weighted quartets. As the dataset comprised both weighted and unweighted quartet sets induced from the same model species tree, we were able to use the unweighted quartets with QFM-F, QFM-FI, and QMC, and weighted quartets with wQMC. Another algorithm that works with weighted quartets is wQFM, but it is excluded from this experiment because wQFM expects a distinct set of weighted quartets while our large simulated datasets contained repeated quartets, albeit with different weights.

In most of the cases QFM-FI outperformed the other methods—among 120 noisy model conditions, QFM-FI was better than all other methods in 70 cases, QMC was better in 20 cases, QFM in 10 cases, and wQMC in only four cases. Among the 30 noiseless model conditions, QFM-FI was better than other methods in four cases, QMC in two cases, QFM in nine cases, and wQMC in seven cases. In five noiseless model conditions, QMC, QFM-F, and QFM-FI were able to perfectly recover the tree (i.e. 0 nRF distance). A more detailed analysis is provided in the Supplementary Material.

From Fig. 1 as well, it is clear that QFM-FI produces trees of superior quality. As *c* increases, all the methods produce better trees. The improvement from QFM-F to QFM-FI is also clearly visible in these graphs. The nRF values in Fig. 1d are higher compared to the other figures because *k *=* *2.6 has been used, instead of 2.8, due to memory constraints—as fewer quartets are analyzed, tree quality has been adversely affected.

From the above experiments, it is clear that QFM-FI produces superior quality trees compared to QFM-F (and QFM). Therefore, in subsequent experiments, we have only run QFM-FI and have not run the other two versions.

#### 3.1.2 Comparison between QFM-FI and QFM-PAUP

To run QFM-PAUP, MSA is given as input to SVDQuartets method in PAUP*, which generates unweighted quartets and finally QFM is used to reconstruct the phylogenetic tree by amalgamating these quartets. The comparison of nRF distance between QFM-FI and QFM-PAUP on 22 different model conditions of the SATe dataset is shown in Supplementary Table S3. QFM-FI performed better in 8 model conditions, while QFM-PAUP was better in 14 model conditions. But only in two model conditions (500L1 and 500M4), the results were statistically significant (Supplementary Table S9). While QFM-PAUP performed better in both of these model conditions, it is noteworthy that QFM-PAUP could not generate tree for some of the replicates of some model conditions (500L1, 500L2, 500L3, 500M2, 500M3, and 500S1). As the details of QFM-PAUP implementation is not available (neither source code, nor any publication), we could not tell whether the failure is a systematic one and for which type of input data might the algorithm fail consistently. QFM-FI, on the other hand, was able to recover a tree in all cases.

#### 3.1.3 Comparison on 37-taxon simulated dataset

**Figure 2.**
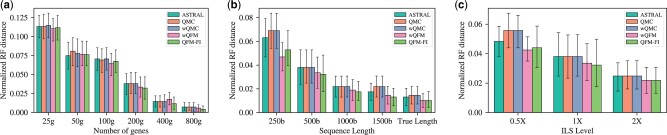
Study on 37-taxon simulated dataset using QFM-FI, wQFM, wQMC, QMC, and ASTRAL by varying number of genes, sequence length, and ILS level. (a) ILS level is moderate and sequence length is 500 bp. (b) ILS level is moderate and number of genes is 200. (c) Number of genes is 200 and sequence length is 500 bp.

We have compared the performance of QFM-FI, QMC, wQMC, wQFM, and ASTRAL on various model conditions in 37-taxon dataset. As a whole, the accuracy of all of these methods was relatively similar. On some model conditions, QFM-FI outperformed ASTRAL, QMC, and wQMC, albeit the differences were small. In one experiment, we varied the number of genes from 25 to 800 using 500 bp sequence length and kept the level of ILS at moderate. The nRF distance for all the methods decreases as the number of gene increases (see Fig. 2a), but QFM-FI outperforms other methods, and the difference between QFM-FI and the other methods, except for wQFM, is statistically significant where number of genes is 200.

Then, we varied the sequence length from 250 bp to its true length using 200 genes and moderate ILS level. All the methods performed well with the increase of the sequence length but QFM-FI is better than other methods (see Fig. 2b). The difference between QFM-FI with QMC, wQMC, and ASTRAL is statistically significant where sequence lengths are 250 and 500 bp. The difference between QFM-FI with QMC and wQMC is also statistically significant where sequence length is 1500 bp.

We subsequently varied the ILS level from high (0.5×) to low (2×) using 200 genes and 500 bp sequence length. In Fig. 2c, 0.5×, 1×, and 2× means high, moderate, and low levels of ILS, respectively. All the methods performed well as the ILS level decreased. The difference between QFM-FI with QMC, wQMC, and ASTRAL is statistically significant at moderate ILS level. All the results of significance tests are provided in the Supplementary Material.

#### 3.1.4 Comparison on 100-taxon simulated dataset

We have compared the tree quality of QFM-FI, wQMC, wQFM, and ASTRAL for 1000 true gene trees over 10 replicates (Supplementary Fig. S3). QMC aborted in this dataset. For all the other methods, the nRF distance was very close to 0, with ASTRAL having the best performance, followed by wQFM, wQMC, and QFM-FI, respectively.

### 3.2 Comparison of methods on biological dataset

#### 3.2.1 Analyses on plant dataset

**Figure 3.**
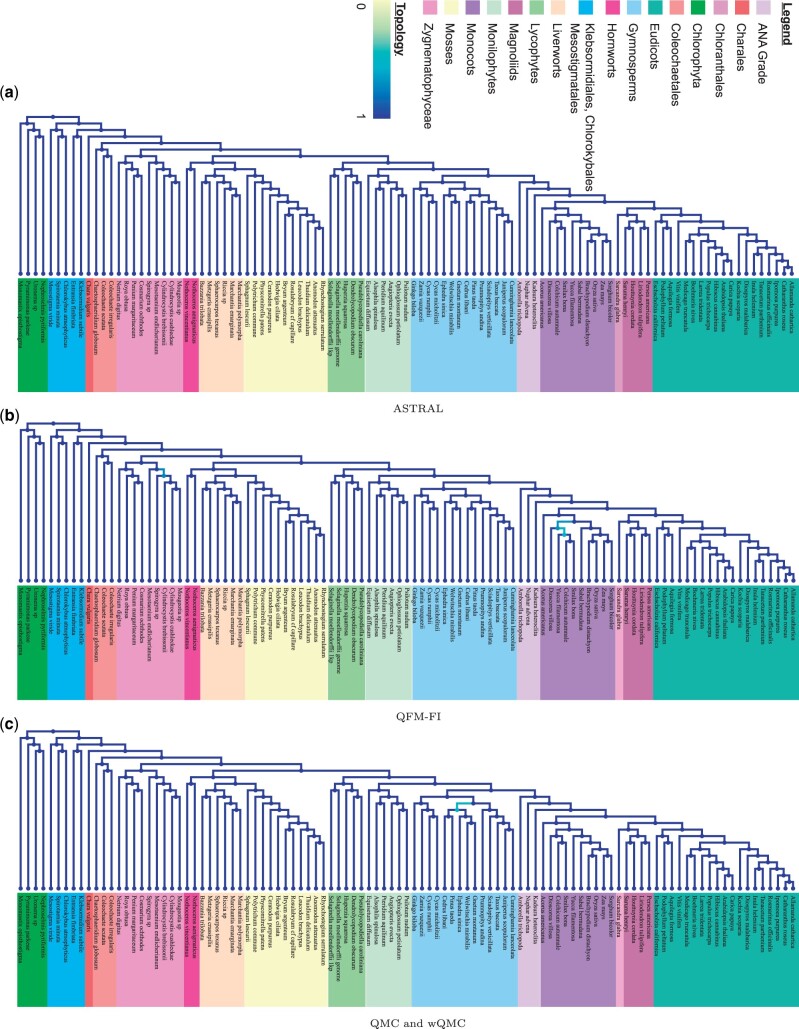
Study of the plant dataset using (a) ASTRAL, (b) QFM-FI, (c) QMC and wQMC. Identical trees are generated by QMC and wQMC. Here, 424 gene trees are used as input. The yellow to blue color scheme, as rendered by Phylo.io (Robinson *et al.* 2016), indicates the similarity of best matching subtrees, with reference to the ASTRAL tree.

Using the 424 gene trees, we have generated weighted and unweighted quartets. We have then fed the unweighted quartets to QFM-FI (Fig. 3b) and QMC (Fig. 3c), and the weighted ones to wQMC (Fig. 3c) to generate the respective species trees. We have also constructed species tree using ASTRAL (Fig. 3a). Notably, QFM of Reaz *et al.* (2014) aborted during execution in this dataset.

All of these methods reconstructed all the major clades, i.e. Eudicots, Chloranthales, Magnoliids, Monocots, ANA Grade, Gymnosperms, Monilophytes, Lycophytes, Hornworts, Liverworts, Mosses, Zygnematophyceae, Coleochaetales, Charales, Klebsormidiales, Chlorokybales, and Mesostigmatales although their internal orientation of leaf nodes are little bit different from each other. QMC and wQMC have generated identical trees. Internal resolution of different clades is given below.

- **Zygnematophyceae.** Branching order of this clade, as recovered by QFM-FI, differs from that of ASTRAL and QMC. But the order recovered by QFM-FI matches with previous DNA sequence based study (Gontcharov 2008).
- **Bryophytes.** All the methods successfully recreated bryophytes, a monophyletic group of non-vascular land plants that includes Bryophyta (mosses), Anthocerotophyta (hornworts), and Marchantiophyta (liverworts) (Nishiyama *et al.* 2004, Goremykin and Hellwig 2005, Wickett *et al.* 2014).
- **Monilophyte and Lycophyte.** The lycophytes and monilophytes have been recovered as successive sister lineages by all the methods, which is also well supported in literature (Pryer *et al.* 2001, Wolf *et al.* 2005, Rai and Graham 2010, Grewe *et al.* 2013, Wickett *et al.* 2014).
- **Gymnosperm.** Gymnosperm clade is recovered and a sister relationship between Gnetales (specified by *Gnetum montanum*) and Pinaceae (specified by *Pinus taeda* and *Cedrus libani*) is reconstructed by all the methods. This reconstruction is congruent with previously reported studies (Burleigh and Mathews 2004, Zhong *et al.* 2010, 2011). The orientation of leaf nodes for this clade in the trees estimated by QMC and wQMC are different with that of the other methods.
- **Angiosperm (flowering plants).** All the estimated trees concluded that *Amborella trichopoda* is the sister species to all other angiosperms. ANA grade, which includes Amborellales (specified by *A.trichopoda*), Nymphaeales (specified by *Nuphar advena*), and Austrobaileyales (specified by *Kadsura heteroclite*), is found to be the subsequent sister lineages of rest of the angiosperms. This finding is in accordance with most prior studies and current phylogenomic assessments of nuclear genes (Jansen *et al.* 2007, Moore *et al.* 2007, Qiu *et al.* 2010, Soltis *et al.* 2011, 1999). In Monocots clade, we have gotten different orientation of leaf nodes for ASTRAL and QFM-FI. But the relationships among magnoliid, monocot, and eudicot clades, as resolved by all the methods, are mostly comparable with existing studies (Moore *et al.* 2010, Soltis *et al.* 2011, Wickett *et al.* 2014).

We also generated unweighted quartets from concatenated alignments of 424 genes using SVDquartets method of PAUP*, which were then amalgamated using QFM-FI, QMC and QFM-PAUP (Supplementary Fig. S4). Each of the resulting species trees failed to recover ANA Grade. They reconstructed magnoliids but misplaced *Sarcandra glabra* and *K.heteroclita* inside magnoliids clade. QMC could not reconstruct monocots clade. Rest of the clades were recovered although their branching order is very different. Overall, for all the methods, quartets decomposed from gene trees resulted in better analysis compared to quartets obtained by SVDquartets from concatenated alignments.

#### 3.2.2 Analyses on avian dataset

**Figure 4.**
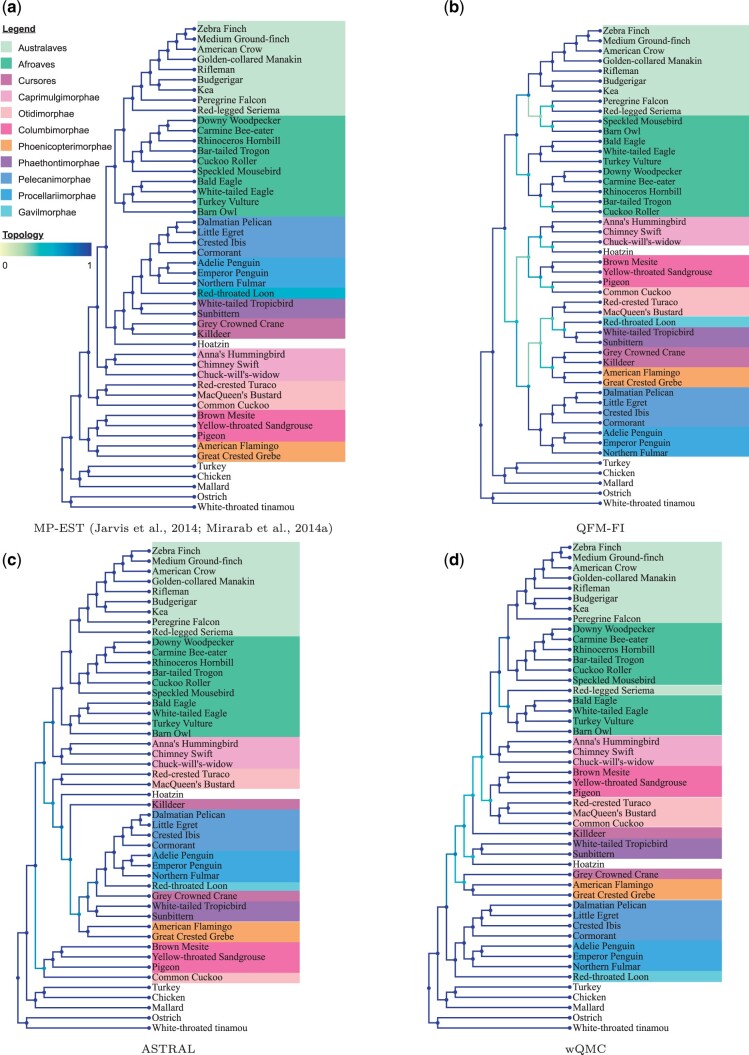
Study of the Avian dataset using QFM-FI, ASTRAL, and wQMC. The yellow to blue color scheme, as rendered by Phylo.io, indicates the similarity of best matching subtrees, with reference to the MP-EST tree. (a) MP-EST (Jarvis *et al.* 2014, Mirarab *et al.* 2014a), (b) QFM-FI, (c) ASTRAL, (d) wQMC.

The trees estimated by QFM-FI, wQMC, and ASTRAL in the avian dataset are shown in Fig. 4. These trees have been compared against the tree estimated by MP-EST that was presented as reference tree in other analyses (Jarvis *et al.* 2014, Mirarab *et al.* 2014a). Notably, even with 64 GB RAM, both QFM of Reaz *et al.* (2014) as well as QMC aborted during execution in this dataset, which contained ∼2.5 billion quartets.

- **Telluraves.** Australaves and Afroaves construct the “core landbirds” or the Telluraves clade (Braun *et al.* 2019). The estimated trees of QFM-FI and ASTRAL recovered Australaves and Afroaves clades to successfully reconstruct Telluraves, albeit with different internal resolution. On the other hand, the estimated tree of wQMC could not cluster Red-legged seriema with other Australaves, thus failing to resolve Australaves clade correctly. Though wQMC could not resolve Australaves and Afroves clades fully, it nevertheless clustered them together. wQFM also recreated all of these relationships, as shown in Mahbub *et al.* (2021).
- **Acquornithia.** Pelecanimorphae, Procellariimorphae, and Gavilmorphae clades construct the “core waterbird” or the Acquornithia clade (Jarvis *et al.* 2014). wQFM (Mahbub *et al.* 2021), ASTRAL, and wQMC resolved all of these small groups as well as the Acquornithia clade. QFM-FI could reconstruct Pelecanimorphae, Procellariimorphae, and Gavilmorphae clades separately but could not resolve the Acquornithia as it put Gavilmorphae (Red-throated loon) in a different clade.
- **Phaethontimorphae.** This clade comprises sunbittern and topicbird (Jarvis *et al.* 2014). wQFM (Mahbub *et al.* 2021), ASTRAL, wQMC, and QFM-FI resolved this clade.
- **Otidimorphae.** This group consists of Red-crested turaco, MacQueen’s bustard, and common cuckoo (Mahbub *et al.* 2021). wQMC recovered this clade but other methods failed to resolve it. This clade was also recovered by wQFM in the analysis performed in Mahbub *et al.* (2021).
- **Caprimulgimorphae (Strisores).** The chuck-will’s-widow (nightjar), swift, and hummingbird form this clade (Braun *et al.* 2019).This clade was recovered by ASTRAL and wQMC. In the study done in Mahbub *et al.* (2021), wQFM also inferred this clade. These species were clustered together into a clade by QFM-FI, albeit it also included hoatzin [the extant species of Opisthocomiformes (Braun *et al.* 2019)] in the same clade. The position of Opisthocomiformes has been highly debated in several assessments (Mayr 2022) and according to several molecular investigations (Hackett *et al.* 2008, Prum *et al.* 2015, Kuhl *et al.* 2021), Opisthocomiformes were found to be near to the Strisores.
- **Columbea.** All of these methods have retrieved Columbimorphae (mesite, sandgrouse, and pigeon) and Phoenicopterimorphae (flamingo and grebe) (Jarvis *et al.* 2014). But none of these methods position them as sister clades and therefore, failed to recover Columbea (flamingo, grebe, pigeon, mesite, and sandgrouse) clade. Although wQFM recovers Columbimorphae and Phoenicopterimorphae, it too is unable to restore Columbea (Mahbub *et al.* 2021).
- **Cursores.** The estimated tree of QFM-FI reconstructed Cursores (grey crowned crane and killdeer) whereas all the other methods in our study, as well as wQFM (Mahbub *et al.* 2021) failed to resolve this sub-group.

There is plenty of proof that estimates of avian phylogeny based on large-scale datasets may be impacted by well-known artifacts (such as long-branch attraction, heterotactic variation, and discordance between gene trees) as well as subtle “data-type effects” that reflect poor fit to empirical data for available models of sequence evolution. The estimations of phylogeny vary depending on whether exons, introns, non-coding ultraconserved elements, conserved non-exonic elements, or TE insertions were employed for analysis (Braun *et al.* 2019). In our analysis, however, we have used the same type of data as input for different quartet-based methods and obtained different trees. This is likely due to the methodological differences of the various quartet-based methods. Even when employing the same collection of gene trees, different approaches may operate under different presumptions and have varying sensitivity to the phylogenetic signal present in the data, thus resolving the complicated relationships at the base of Neoaves more or less effectively.

The analyses on Amniota and Angiosperm datasets are provided in the Supplementary Material.

### 3.3 Running time comparison

We have compared the running time of different variants of QFM with QMC, wQMC, and ASTRAL. In several datasets, we originally had gene trees which needed to be pre-processed to generate quartets to be consumed by QFM, QMC, and wQMC. We note that this pre-processing time is not included in the running time comparisons.

#### 3.3.1 Running time comparison of QFM and QFM-F

**Figure 5.**
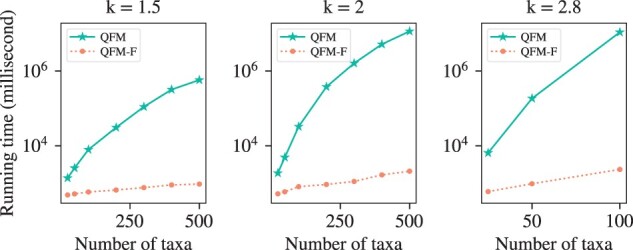
Running time comparison of QFM and QFM-F (c=70%).

For running time analysis, we varied *n* and *k* while keeping *c* fixed at 70%. We have used k∈{1.5,2,2.8}. For model conditions where *k* is set to either 1.5 or 2, we have varied *n* from 25 to 500. Since QFM does not scale to large datasets, we have varied number of taxa from 25 to 100 for the model conditions, where *k* is set to 2.8. We have taken the average running time of 20 replicates for each model condition. Figure 5 shows the running time comparison of QFM and QFM-F where it is very clear that QFM does not scale to large datasets. But we have been able to significantly improve its performance, as observed from the QFM-F curves, thus enabling it to be used in larger phylogenetic studies.

#### 3.3.2 Running time comparison of QFM-F, QFM-FI, QMC, and wQMC

The running time curves are shown in Supplementary Fig. S5. The running time of QFM-F and QFM-FI are very similar. Thus, we were able to achieve tree quality improvement with a little sacrifice of the running time improvements that we incorporated into QFM. For example, the largest difference in running time between QFM-F and QFM-FI was <25 min when number of taxa was as high as 2000, with 382 540 999 quartets (*k *=* *2.6) and *c* was 70%.

The running time of QFM-F and QFM-FI is better than that of QMC and wQMC when the quartet count is quadratic in the number of taxa. But running time of QFM-F and QFM-FI becomes higher than that of QMC and wQMC when *k* is 2.8. It can also be observed that the running time of all the methods increase while number of taxa, quartet or consistency level increases. Nevertheless, the running time of all the methods stay reasonable for quite large datasets.

#### 3.3.3 Running time comparison of QFM-F, QFM-FI, and QFM-PAUP

We have compared the running time of QFM-F, QFM-FI, and QFM-PAUP on different model conditions from the SATe dataset. QFM-PAUP is significantly faster than QFM implementation of Reaz *et al.* (2014). In fact, it is faster than QFM-F and QFM-FI when the number of taxa is 100. But when 500 taxa datasets were used, QFM-F and QFM-FI significantly outperformed QFM-PAUP in terms of running time. This is shown in Supplementary Fig. S6. Interestingly, in all of these experiments, QFM-FI demonstrated faster running time than QFM-F.

#### 3.3.4 Running time comparison on 37-taxon simulated dataset

The running time comparisons among QFM-FI, QMC, wQMC, and ASTRAL for varying number of genes, ILS, and gene lengths are shown in Supplementary Fig. S7. Since it is a small dataset, the running time for all the method is very low. In these model conditions, QFM was slower than the other methods, albeit the largest running time for QFM was 156 s only. For the input of wQMC, the quartets were pre-processed to produce a distinct set of quartets with their frequency (number of appearances of same topology) as weight. This significantly reduced the input size, which led to the very small running time for wQMC.

As the number of genes increased, the running time of ASTRAL, QMC, and QFM-FI increased as well which is expected because number of quartets also increases with the increase of number of genes. As the level of ILS dropped, so did the running times of ASTRAL and QFM-FI, which was also expected but the running time of QMC remained fairly unchanged. On the other hand, as the sequence length increased, the running time of ASTRAL and QFM-FI decreased, while that of QMC remained relatively unchanged. For true sequence length, the running time of QFM-FI increased a little bit. As the number of genes is 500 in the last two experiments (decreasing ILS level and increasing sequence length), the number of generated quartets is nearly equal. As a result, the running time of QMC has remained relatively constant.

#### 3.3.5 Running time comparison on 100-taxon simulated dataset

Although tree quality of ASTRAL (Supplementary Fig. S3) was good in this experiment, running time is quite high compared to other methods (Supplementary Fig. S8). The running time of QMC and wQMC was better in this dataset than ASTRAL and QFM-FI.

### 3.4 Discussion

In this work, we have made several changes to the QFM algorithm so as to significantly improve its running time as well as the quality of estimated trees. Named as QFM-FI, our method has been analyzed theoretically and we have been able to provide bounds on the time and space complexity of our algorithm. We have improved the running time of the MFM bi-partitioning phase by a factor of $O(n^{3})$. The overall time and space complexity of QFM-FI has been shown to be $O(\max(nm^{2},n^{3}m))$ and O(m+n), respectively.

We have carried out a thorough comparison of QFM-FI with existing state-of-the-art supertree methods on simulated and biological datasets, which cover a wide range of model conditions. We have observed a significant improvement in generated tree quality as well as in speed. QFM-FI outperformed all other approaches in 70 cases out of 120 noisy model conditions and 4 cases out of 30 noiseless model conditions of simulated dataset-1. The running time of QFM-FI is better than QMC and wQMC when *k* is 1.5 or 2. On 37-taxon simulated dataset, out of 12 model conditions, QFM outperformed ASTRAL in two cases, QMC in three cases, and wQMC in three cases with statistical significance. When we have averaged the nRF distance over 20 replicates for each of these 12 model conditions, it is observed that QFM outperformed ASTRAL, QMC, and wQMC on all of these 12 model conditions although only some of the differences of these averages between QFM-FI and other methods are statistically significant. We have also noted that running time of QFM-FI is a little bit slow in comparison with ASTRAL, QMC, and wQMC although the largest running time is <3 min. Also, we observed that when there is numerous amount of redundant quartet trees in input, QFM-FI is more memory efficient than QMC–QMC aborted on the whole quartet set of 100-taxon simulated dataset (see Supplementary Material for the details of memory comparison).

In biological datasets, we have observed that the estimated tree of QFM-FI is quite comparable and consistent with the biological beliefs. QFM-FI is more congruent with the biological notions than the other methods on avian dataset. The variability at the base of the Neoaves, as noted in Braun *et al.* (2019), is consistent with the complexity of the group and the difficulties in accurately reconstructing its phylogeny. This variability may be due in part to the types of data used in the analyses, as different data types can provide conflicting signals and make it difficult to accurately resolve relationships (Reddy *et al.* 2017, Braun and Kimball 2021). It is also possible that the variability reflects simultaneous speciation events, as proposed by Suh (2016). It is important to consider these issues when interpreting the results of phylogenetic analyses of the Neoaves clade and to continue to explore and test different approaches to improve our understanding of the evolutionary relationships within this group. It is also important to note that the differences among trees at the base do not necessarily indicate a problem with the quartet assembly method used, but rather reflect the inherent complexity of the problem and the challenges in accurately reconstructing the phylogeny of Neoaves. The fact that the trees obtained by different methods are quite different on the avian dataset suggests that empirical phylogeneticists should attempt a variety of quartet approaches rather than just the widely used ASTRAL method. For example, If QFM-FI and ASTRAL provide comparable or identical results, it should increase the trust in the conclusions (which is the case for plant dataset).

On other biological datasets, the estimated trees of QFM-FI, ASTRAL, QMC, and wQMC are quite similar and likewise compatible with the biological concepts. We have also observed that QFM of Reaz *et al.* (2014) is not scalable to large biological datasets, such as plant and avian dataset. QMC also failed to estimate phylogenetic tree on avian dataset whereas other state-of-the-art methods as well as QFM-FI could.

While an existing tool, PAUP*, does have a fast implementation of QFM, which can be used by biologists, the inner workings of that implementation is not available in published literature. Our work, on the other hand, clearly describes the changes made to QFM which led to the improved running time as well as improved tree quality. We have also shown that QFM-FI runs faster than QFM-PAUP when the study contains larger number of taxa (see results for 500 taxa versus 100 taxa in Supplementary Fig. S6). Last but not the least, PAUP* does not allow for any other methods to generate the quartets except for SVDQuartets, before phylogeny reconstruction can proceed with QFM. With QFM-FI, on the other hand, any tool can be used in the upstream analysis to generate the set of quartets. This is an important point, especially because our analysis of plant dataset indicated that quartets generated from gene trees in the upstream analysis may be more reliable than ones generated from SVDquartets method. Therefore, we believe that the results in this article adds considerable contribution in the field of computational phylogenetics.

Overall, through our work, we have been able to significantly improve the scalability of QFM. We have also shown that the tool is still very much relevant in today’s phylogenetic data analysis, as it produces trees with comparable quality with state-of-the-art methods in different simulated as well as biological datasets.

## 4 Conclusion

In this article, we have presented an improved and scalable version of QFM algorithm and have demonstrated that it performs better or comparably over several state-of-the-art algorithms. Our improved version of QFM, named QFM-FI, has been assessed through an extensive simulation study. We have then used QFM-FI to analyze several biological datasets. QFM-FI was able to recover phylogenetic relationships well in the simulated as well as the biological datasets, while QFM of Reaz *et al.* (2014) and SVDquartets+PAUP* were unable to do so on several datasets. For datasets with a large set of quartets, QMC, the widely used quartet-based phylogeny reconstruction method, also failed. We have provided time and memory complexity analysis of QFM-FI, which should draw confidence in its scalability. We hope that the results presented in this study will encourage the biologists to use the tool in their phylogenomic studies and reach their goals of resolving phylogenetic relationships of many species successfully.

## Supplementary Material

btad332_Supplementary_Data
