# Supplementary material for: Quartet Fiduccia–Mattheyses revisited for larger phylogenetic studies
Source: Bioinformatics. 2023 Jun 7;39(6):btad332. doi: 10.1093/bioinformatics/btad332 (PMC10260390; doi:10.1093/bioinformatics/btad332)
Supplement: btad332_Supplementary_Data [file btad332_supplementary_data.pdf]

Supplementary Material to Quartet Fiduccia-Mattheyses Revisited for Larger Phylogenetic Studies

SHARMIN AKTER MIM, MD. ZARIF-UL-ALAM, REZWANA REAZ, MD. SHAMSUZZOHA BAYZID, M. SAIFUR RAHMAN

1. OVERVIEW

This supplementary document contains additional data, results and further details of the *Quartet Fiduccia-Mattheyses Fast and Improved* (QFM-FI) algorithm.

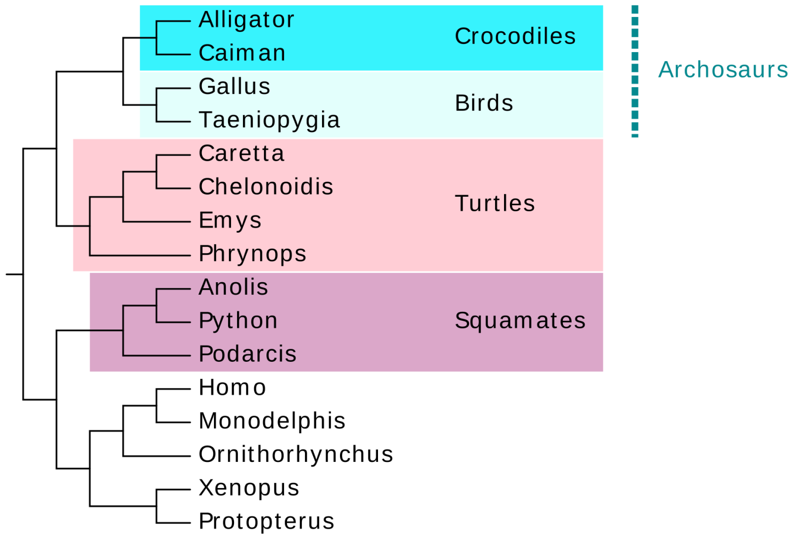

Fig. S1. Study of the amniota dataset using QFM-FI, QMC, wQMC and ASTRAL. All the methods estimated identical phylogenetic tree.

2. TOOLS

The QFM implementation of [1] was obtained from the authors through private communication. We have made it publicly available at https://github.com/sharmin-mim/qfm_java/tree/master/qfm_reaz_et_al. For running QMC and wQMC, we have used Quartet MaxCut version 3.0 [2]. We have used ASTRAL-III (version 5.7.3) [3]. PAUP* has been downloaded from https://paup.phylosolutions.com/. Last but not the least, our fast and improved re-implementation of QFM can be found here: https://github.com/sharmin-mim/qfm_java.

For data visualization, we have used Matplotlib [4], pandas [5] and seaborn [6]. EvolView [7] and Phylo.io [8] have been used for phylogenetic tree visualization, annotation and comparison.

For most of our experiments, we have used a laptop with Intel(R) Core(TM) i7-12700H CPU @ 4.70 GHz x 20, 16 GB RAM and Ubuntu 22.04 OS. As more memory was required for the experiments conducted on SATe, Plant and Avian

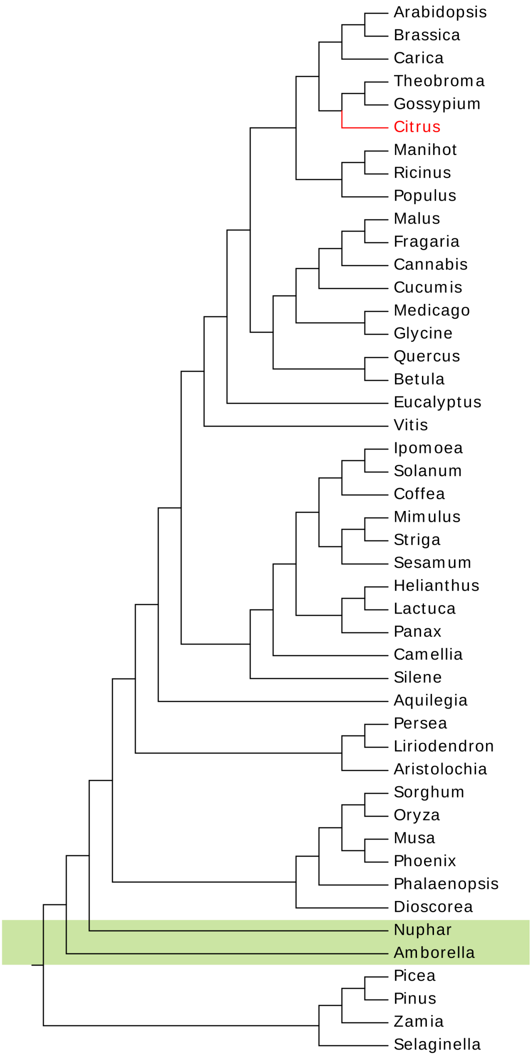

(a) QFM-FI and ASTRAL

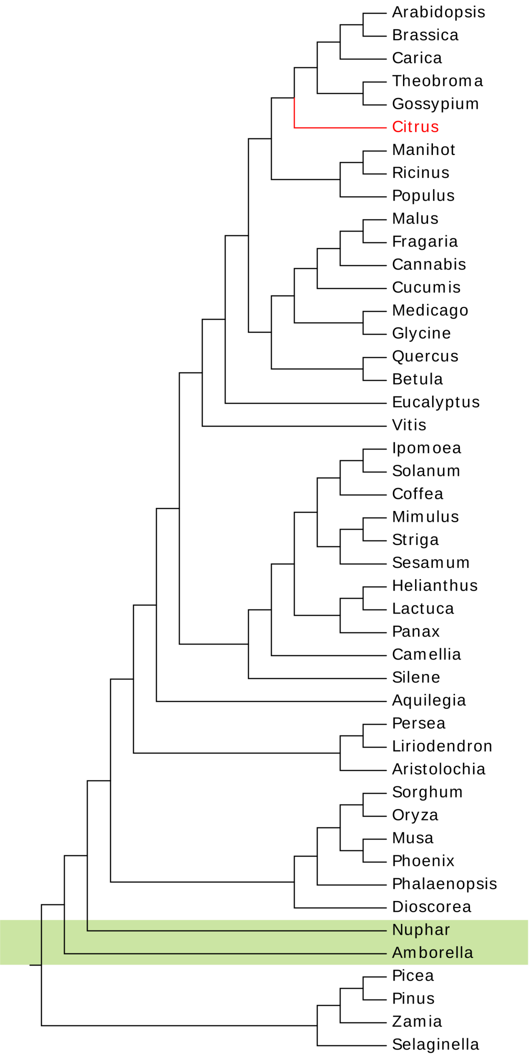

(b) QMC and wQMC

Fig. S2. Study of the angiosperm dataset using QFM-FI, QMC, wQMC and ASTRAL. All the methods classified *Amborella* as a sister to Nymphaeales (shown in green shaded area) and the other angiosperms.

datasets, we used a machine with 64 GB RAM. For the experiments of Fig. 1c and Fig. 1d of main manuscript, 64 GB RAM was not enough and hence we had to use a machine with 252 GB RAM.

3. TREE COMPARISON METRICS

In the simulated datasets, the reference trees are available; therefore comparison can be performed in terms of normalized Robinson Foulds Distance (nRF). The Robinson Foulds (RF) distance can be defined as the sum of False Negative (FN) and False Positive (FP) edges [9]. An edge in the reference tree is referred to as false negative edge if it is not found in the estimated tree. A false positive edge, on the other hand, is an edge in the estimated tree that does not occur in the reference tree. RF distance can range between 0 to $2n-6$, where n is the number of leaf nodes in each tree [10]. It can thus be normalized through division by $2n-6$. In other words, $nRF = \frac{RF\ distance}{2n-6}$.

In the biological datasets, on the other hand, no model tree is available. Therefore, the estimated trees were analyzed based on existing literature and biological beliefs.

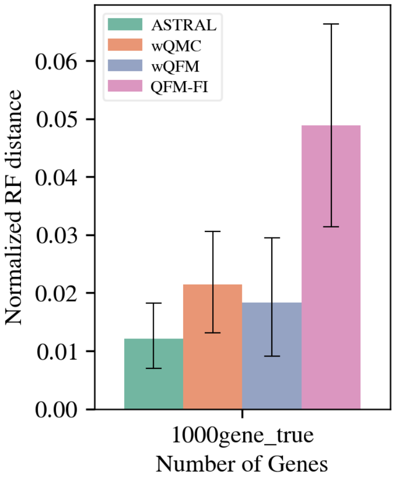

Fig. S3. Study on 100-taxon simulated dataset using QFM-FI, wQFM, wQMC and ASTRAL.

Algorithm S1 QFM

Input: Q: Set of quartets
Output: T: Phylogenetic tree

Read the input quartets and count their frequencies
Find out set of Taxa P
T $\leftarrow$ MODIFIED_SQP(Q, P)
return T

Algorithm S2 Function for Modified_SQP

Function MODIFIED_SQP(Q, P)
  if $P = \emptyset$ **then return** null
  else if $Q = \emptyset$ or $Size[P] \leq 3$ **then return** depth one tree
  else
    $(P_l, P_r) \leftarrow$ INITIAL_PARTITION(Q, P)
    $(P_l, P_r) \leftarrow$ MFM_PARTITION(Q, P_l, P_r)
    ADD dummy taxon t_d in P_l and P_r
    for each deferred quartet $q_d \in$ Q **do**
      if All 4 taxa of q_d are in P_l (P_r) **then**
        PUT q_d in Q_l (Q_r)
      else
        PUT q_d in Q_l (Q_r) if the deserted taxon is in P_r (P_l), after replacing the deserted taxon with t_d, and update its frequency
      end if
    end for
    $T_l \leftarrow$ MODIFIED_SQP(Q_l, P_l)
    $T_r \leftarrow$ MODIFIED_SQP$((Q_r, P_r)$
    $T \leftarrow$ MERGE(T_l, T_r)
  end if
  return T
end Function

4. SPACE COMPLEXITY ANALYSIS

The memory requirements of QFM-FI can be analyzed as follows. Let, n and m be the cardinality of taxa set P and quartet set Q respectively.

- Since hashing is used at the time of storing and counting the frequency of each quartet, the required space for that step is $\mathcal{O}(m)$.

Algorithm S3 Function for Initial_Partition

```
Function INITIAL_PARTITION(Q, P)
    P_l = ∅
    P_r = ∅
    SORT Q in descending order of quartet frequency
    for each quartet q((t_1, t_2), (t_3, t_4)) ∈ Q (in sort order) do
        for each taxa t_i of q do
            CHECK which Partition t_i is assigned to
        end for
        if each t_i is in unassigned taxa set P_u then
            P_l ← P_l ∪ t_1 ∪ t_2
            P_r ← P_r ∪ t_3 ∪ t_4
        else
            if t_1 ∈ P_u then
                if t_2 ∉ P_u then
                    PUT t_1 in the same partition as t_2
                else if t_3 ∉ P_u then
                    PUT t_1, t_2 in the opposite partition of t_3
                else
                    PUT t_1, t_2 in the opposite partition of t_4
                    PUT t_3 in the same partition as t_4
                end if
            end if
            if t_2 ∈ P_u then
                PUT t_2 in the same partition as t_1
            end if
            if t_3 ∈ P_u then
                if t_4 ∉ P_u then
                    PUT t_3 in the same partition as t_4
                else
                    PUT t_3, t_4 in the opposite partition of t_1
                end if
            end if
            if t_4 ∈ P_u then
                PUT t_4 in the same partition as t_3
            end if
        end if
    end for
    return (P_l, P_r)
end Function
```

- The initial bi-partitioning step comprises sorting Q and bi-partitioning P (see Algorithm S3). Sorting the quartets require $\mathcal{O}(m)$ space, while the space needed for bi-partitioning P is $\mathcal{O}(n)$. So, the initial bi-partitioning step takes $\mathcal{O}(m+n)$ memory.
- In MFM partitioning (see Algorithm 1 of main manuscript), relevant quartet indices of each taxon is stored. As a quartet is formed by 4 distinct taxa, its index will be relevant to each of those taxa and thus be stored 4 times. Therefore, the space needed for maintaining the relevant quartet indices is $\mathcal{O}(m)$. P is also bifurcated and total space for these two partition is n. So, overall needed space for MFM partitioning is $\mathcal{O}(m+n)$.
- Short quartet puzzle step (see Algorithm S2) contains a set of quartets Q and a set of taxa P. Q and P both are bifurcated for next recursive short quartet puzzle steps. Before participating into next recursive call, Q and P are deleted and only newly bifurcated parts remain. So total required space for this step is $\mathcal{O}(m+n)$.

Therefore, overall space complexity of QFM-FI is $\mathcal{O}(m+n)$.

5. COMPARISON AMONG QMC, WQMC, QFM AND QFM-FI ON SIMULATED DATASET-I

As mentioned in the main manuscript, we have conducted extensive experiments to compare QMC, wQMC, QFM and QFM-FI. Based on the results shown in Tables S1 and S2 some comparisons can be done as follows.

- **QFM-FI vs. QFM.** QFM-FI is better than QFM (as well as QFM-F) in 84 noisy model conditions; the improvement is statistically significant in 59 of those conditions. On the contrary, QFM is better than QFM-FI in only 18 noisy model conditions and of those only 2 are statistically significant. As to the noiseless models, QFM-FI is better than QFM in 5 cases, however, none of them are statistically significant. QFM is better than QFM-FI in 10 noiseless model conditions, 1 of which is statistically significant. Notably, for statistical significance testing in all the model conditions, we have used Wilcoxon signed-rank test (with $\alpha = 0.05$) and all the test results are available later in this file.
- **QFM-FI vs. QMC.** QFM-FI produced superior quality trees compared to QMC in 78 noisy model conditions, 51 of which were statistically significant. QMC, on the other hand, performed better than QFM-FI in 18 noisy model conditions, albeit only 2 of those were statistically significant. QFM-FI performed better than QMC in 18 noiseless model conditions, 12 of which are statistically significant. QMC results were better in 4 noiseless model conditions; only 1 of these was statistically significant.
- **QFM-FI vs. wQMC.** QFM-FI is better than wQMC in 97 noisy model conditions (77 results are statistically significant). On the other hand, wQMC results were better in 13 noisy model conditions, only 2 of these being statistically significant. In case of the noiseless model conditions, QFM-FI was superior in 18 cases (14 of those are statistically significant). In contrast, wQMC was better than QFM-FI in 13 noiseless model conditions but only 2 of these conditions are statistically significant.

6. COMPARISON BETWEEN QFM-FI AND QFM-PAUP

Table S3 illustrates the comparison of normalized RF distance between QFM-FI and QFM-PAUP on 22 different model conditions of the SATe dataset.

7. COMPARISON OF METHODS ON BIOLOGICAL DATASET

A. Analyses on Amniota dataset

We have re-analyzed the amniota dataset (amino acid (AA) gene trees) from Chiari et al. [11] with an aim of figuring out the position of turtles (Caretta, Chelonoidis, Emys, Phrynops) relative to birds (Gallus, Taeniopygia) and crocodiles (Alligator, Caiman). It is suggested by previous research [11–14] that there is a sister relationship between birds and crocodiles (forming archosaurs), as well as a sister relationship between turtles and archosaurs. Turtles were correctly classified as a sister clade to archosaurs by QFM-FI as well as QMC, wQMC and ASTRAL (see Figure S1). This relationship was also supported by wQFM method in [15].

B. Analyses on Angiosperm dataset

We have analyzed the angriosperm dataset from Xi et al. [16]. The central concern of this study is to find out the position of *Amborella trichopoda Baill*. QFM-FI, QMC, wQMC and ASTRAL place *Amborella* as a sister to *Nymphaeales* and rest of the angiosperms. According to various hypotheses [16–18], the first lineage of existent angiosperms was *Amborella* plus *Nuphar*. All the estimated trees supported these hypotheses (see Figure S2). The location of Sapindales (*Citrus*) distinguishes QFM-FI and ASTRAL from QMC and wQMC on one edge which is shown in red color in Figures S2a and S2b. The tree reconstructed by QFM-FI is identical with the wQFM tree in [15].

8. WILCOXON SIGNED-RANK TEST

In order to compute the statistical significance of differences between QFM (QFM-F or QFM-FI) and other methods, we have used Wilcoxon signed-rank test (with $\alpha = 0.05$) on various model conditions of different datasets and they are shown in tables S4 to S12.

9. QUARTET SCORE

As we do no have true tree for biological datasets, we have calculated quartet scores on different biological datasets which is shown in Table S13.

10. MEMORY COMPARISON

We have done a memory analysis on the simulated dataset 1. In this analysis, we have varied the number of taxa from 25 to 500. From Fig. S9, we see that wQMC and QMC are more memory efficient than QFM-F and QFM-FI.

Then, we have done a memory analysis of QFM-FI, QMC, wQMC and ASTRAL on various model conditions of 37-taxon dataset. In this case, 20 replicates of each model conditions are used. In Fig. S10, it is seen that as the number of genes increases, peak memory of QMC, wQMC, ASTRAL also increases because the number of quartets also increases

Table S1. Comparison of QMC, wQMC, QFM-F and QFM-FI under various noisy model conditions. Best results are shown in bold. $\{w|\epsilon\}qmc$: Left column represents data for wQMC, right column for QMC. $qfm - \{f|fi\}$: Left column represents data for QFM-F, right column for QFM-FI.

n	*k*	***Normalized RF Distance of 20 Replicates***															
		Correct = 70%				*Correct = 80%*				*Correct = 90%*				*Correct = 95%*			
		$\{w\|\epsilon\}qmc$		$qfm - \{f\|fi\}$		$\{w\|\epsilon\}qmc$		$qfm - \{f\|fi\}$		$\{w\|\epsilon\}qmc$		$qfm - \{f\|fi\}$		$\{w\|\epsilon\}qmc$		$qfm - \{f\|fi\}$	
25	1.5	.920	**.914**	.959	.951	.855	**.732**	.755	.755	.661	.672	**.617**	.657	.481	.482	**.447**	**.447**
25	2	.411	.321	.322	**.287**	.217	.204	.219	**.183**	**.120**	.123	.157	.126	.081	**.051**	.076	.054
25	2.8	.007	**0**	.045	**0**	**0**	**0**	.014	**0**	**0**	**0**	**0**	**0**	**0**	**0**	**0**	**0**
50	1.5	**.969**	.973	.973	.976	.906	**.863**	.878	.875	.781	**.735**	.752	.753	.710	**.685**	.703	.711
50	2	.512	**.462**	.508	.469	.329	.327	.334	**.324**	.238	.221	.225	**.219**	.200	**.184**	**.184**	**.184**
50	2.8	.011	.006	.072	**.004**	.002	**.001**	.040	.002	**0**	**0**	.017	**0**	**0**	**0**	.006	**0**
100	1.5	**.983**	.984	.991	.993	.950	.949	.949	**.948**	.890	.868	.865	**.856**	.846	.832	**.827**	.830
100	2	.603	.583	.607	**.571**	.500	**.474**	.497	.491	.338	.337	.333	**.332**	**.290**	.305	.302	.295
100	2.8	.014	**.009**	.080	.010	.004	**.002**	.045	**.002**	**.001**	**.001**	.021	**.001**	.001	**0**	.010	.001
200	1.5	**.992**	.996	.998	.997	.974	.975	.967	**.963**	.933	.928	.902	**.898**	.915	.905	**.859**	**.859**
200	2	.722	.700	.709	**.695**	.626	**.573**	.598	.585	.503	.505	.497	**.491**	**.424**	.431	.433	**.424**
200	2.8	.027	.013	.087	**.012**	.020	**.003**	.056	.004	.015	**.001**	.028	**.001**	.003	**0**	.016	**0**
300	1.5	.997	**.996**	.999	.999	.989	.977	**.972**	.974	.971	.952	.925	**.922**	.960	.940	**.903**	**.903**
300	2	.790	**.775**	.785	**.775**	.680	.680	.683	**.674**	.597	.587	**.583**	**.583**	**.522**	.538	.528	.526
300	2.8	.018	.013	.095	**.012**	.008	.004	.065	**.003**	.016	**.001**	.028	**.001**	.014	**.001**	.016	**.001**
400	1.5	**.994**	.997	**.994**	**.994**	.978	.979	**.974**	**.974**	.954	.958	**.950**	**.950**	.942	.950	**.931**	**.931**
400	2	.798	.790	.794	**.780**	.727	.722	.710	**.707**	.645	.645	**.631**	.633	.597	.603	.593	**.589**
400	2.8	.027	.020	.109	**.019**	.007	**.003**	.066	.004	.011	**.001**	.029	**.001**	.007	**0**	.017	**0**
500	1.5	.995	.993	.993	**.990**	.981	.979	.972	**.971**	.963	.962	**.948**	.949	.950	.950	**.929**	.930
500	2	.851	**.842**	.845	**.842**	.783	.768	.759	**.748**	.696	.693	.671	**.669**	.655	.662	.648	**.644**
500	2.8	.030	**.020**	.107	**.020**	.015	**.005**	.066	**.005**	.011	.002	.034	**.001**	.006	**0**	.014	**0**
800	1.5	.997	.991	.990	**.988**	.983	.982	.970	**.969**	.975	.968	.948	**.946**	.965	.963	.936	**.933**
800	2	.866	.872	.863	**.854**	.811	.810	.789	**.784**	.748	.745	.719	**.716**	.708	.710	.686	**.683**
800	2.8	.031	**.020**	.105	**.020**	.013	**.008**	.070	**.008**	.009	**.002**	.033	**.002**	.005	**.001**	.019	**.001**
1000	1.5	.998	.993	.992	**.989**	.990	.986	.978	**.976**	.981	.977	**.960**	.961	.974	.973	**.948**	**.948**
1000	2	.894	.889	.881	**.878**	.843	.838	.822	**.817**	.789	.785	.763	**.759**	.749	.754	**.726**	**.726**
2000	1.5	.997	.993	.992	**.990**	.992	.989	.979	**.978**	.987	.983	.968	**.967**	.981	.976	**.957**	**.957**
2000	2	.918	.918	.910	**.905**	.890	.888	.868	**.866**	.851	.850	.823	**.821**	.821	.832	.798	**.797**
3000	1.5	.998	.994	.993	**.992**	.996	.991	.984	**.983**	.992	.987	.971	**.970**	.989	.981	**.961**	**.961**
3000	2	.940	.934	.927	**.922**	.916	.912	.891	**.887**	.885	.879	.846	**.845**	.861	.861	**.823**	**.823**

Table S2. Comparison of QMC, wQMC, QFM-F and QFM-FI under various noiseless model conditions. Best results are shown in bold. The star marked values look identical with the bold ones due to rounding.

n	*k*	***Normalized RF Distance of 20 Replicates***			
		Correct = 100%			
		wQMC	***QMC***	***QFM-F***	***QFM-FI***
25	1.5	.452	**.397**	.512	.477
	2	.044	.059	.046	**.039**
	2.8	**0**	**0**	**0**	**0**
50	1.5	.643	**.632**	.640	.650
	2	**.141**	.149	.151	.151
	2.8	**0**	**0**	**0**	**0**
100	1.5	.769	.792	**.758**	.768
	2	**.234**	.258	.257	.255
	2.8	.001	**0**	**0**	**0**
200	1.5	.865	.865	**.829**	.830
	2	**.360**	.395	.398	.398
	2.8	**0**	**0**	**0**	**0**
300	1.5	.930	.923	**.880**	.881
	2	**.496**	.518	.506	.511
	2.8	.006	**0**	**0**	**0**
400	1.5	.919	.937	**.906**	.906*
	2	**.540**	.557	.549	.551
	2.8	.007	**0**	**0**	**0**
500	1.5	.930	.945	**.911**	.912
	2	**.590**	.607	.596	.599
	2.8	.004	**0**	**0**	**0**
800	1.5	.947	.948	.908*	**.908**
	2	.654	.682	**.652**	.654
	2.8	.004	**0**	**0**	**0**
1000	1.5	.965	.962	.931	**.930**
	2	**.691**	.719	.691*	.691*
2000	1.5	.972	.972	**.947**	.947*
	2	.790	.808	**.771**	.772
3000	1.5	.981	.972	.947	**.946**
	2	.827	.838	**.795**	.795*

Table S3. Comparison of tree quality between QFM-FI and QFM-PAUP on different model conditions of SATe dataset. Best results are shown in bold.

Model condition	*No. of quartets*	*Normalized RF distance*	
		QFM-FI	*QFM-PAUP*
100L1	3921225	.2231	**.2198**
100L2	3921225	**.1631**	.1641
100M1	3921225	.2263	**.2253**
100M2	3921225	**.1990**	.2011
100M3	3921225	**.1714**	.1719
100S1	3921225	**.2230**	.2250
100S2	3921225	**.1446**	.1467
500L1	36067497	.2919	**.2870**
500L2	36067497	.2702	**.2697**
500L3	36067497	.2759	**.2707**
500L4	36067497	.2134	**.2108**
500L5	36067497	.2125	**.2115**
500M1	36067497	.3030	**.3021**
500M2	36067497	.2907	**.2898**
500M3	36067497	.2814	**.2769**
500M4	36067497	.2092	**.2063**
500M5	36067497	.2132	**.2116**
500S1	36067497	**.3020**	.3045
500S2	36067497	**.2802**	.2805
500S3	36067497	.2825	**.2799**
500S4	36067497	**.2120**	.2124
500S5	36067497	.2137	**.2110**

with the increase of number of genes. But for QFM-FI, peak memory remains almost the same because the number of taxa is the same. In Fig. S11 and Fig. S12, the peak memory of QMC, wQMC and ASTRAL remains almost the same for different model conditions because the number of genes is fixed at 200 and number of quartet is almost same for fixed number of genes. It is also observed that the peak memory of QFM-FI decreases as the sequence length increases (Fig. S11) or as the ILS level decreases (Fig. S12). The number of quartet trees of same topology generated from gene trees increases with the increase of sequence length or with the decrease of ILS level. Thus, the size of distinct quartet set reduces and the peak memory usage of QFM-FI shrinks as well. If the quartet set of a dataset contains numerous redundant quartets (quartets of same topological structure), in that case QFM-FI will be more memory efficient than QMC.

REFERENCES

1. R. Reaz, M. S. Bayzid, and M. S. Rahman, "Accurate Phylogenetic Tree Reconstruction from Quartets: A Heuristic Approach," PLOS ONE **9**, 1–13 (2014).
2. E. Avni, R. Cohen, and S. Snir, "Weighted Quartets Phylogenetics," Syst. Biol. **64**, 233–242 (2014).
3. C. Zhang, M. Rabiee, E. Sayyari, and S. Mirarab, "ASTRAL-III: polynomial time species tree reconstruction from partially resolved gene trees," BMC bioinformatics **19**, 15–30 (2018).
4. J. D. Hunter, "Matplotlib: A 2D graphics environment," Comput. Sci. & Eng. **9**, 90–95 (2007).

5. W. McKinney *et al.*, "Data structures for statistical computing in python," in *Proceedings of the 9th Python in Science Conference,* vol. 445 (Austin, TX, 2010), pp. 51–56.
6. M. L. Waskom, "seaborn: statistical data visualization," J. Open Source Softw. **6**, 3021 (2021).
7. B. Subramanian, S. Gao, M. J. Lercher, S. Hu, and W.-H. Chen, "Evolview v3: a webserver for visualization, annotation, and management of phylogenetic trees," Nucleic Acids Res. **47**, W270–W275 (2019).
8. O. Robinson, D. Dylus, and C. Dessimoz, "*Phylo.io* : Interactive Viewing and Comparison of Large Phylogenetic Trees on the Web," Mol. Biol. Evol. **33**, 2163–2166 (2016).
9. D. F. Robinson and L. R. Foulds, "Comparison of phylogenetic trees," Math. biosciences **53**, 131–147 (1981).
10. C. R. Linder and T. Warnow, "An Overview of Phylogeny Reconstruction," in *Handbook of Computational Molecular Biology,* S. Aluru, ed. (CRC Press, 2005), chap. 19.
11. Y. Chiari, V. Cahais, N. Galtier, and F. Delsuc, "Phylogenomic analyses support the position of turtles as the sister group of birds and crocodiles (Archosauria)," BMC biology **10**, 65 (2012).
12. S. Mirarab, M. S. Bayzid, and T. Warnow, "Evaluating Summary Methods for Multilocus Species Tree Estimation in the Presence of Incomplete Lineage Sorting," Syst. Biol. **65**, 366–380 (2016).
13. A. F. Hugall, R. Foster, and M. S. Y. Lee, "Calibration choice, rate smoothing, and the pattern of tetrapod diversification according to the long nuclear gene RAG-1," Syst. biology **56**, 543–563 (2007).
14. N. Iwabe, Y. Hara, Y. Kumazawa, K. Shibamoto, Y. Saito, T. Miyata, and K. Katoh, "Sister Group Relationship of Turtles to the Bird-Crocodilian Clade Revealed by Nuclear DNACoded Proteins," Mol. Biol. Evol. **22**, 810–813 (2004).
15. M. Mahbub, Z. Wahab, R. Reaz, M. S. Rahman, and M. S. Bayzid, "wQFM: highly accurate genome-scale species tree estimation from weighted quartets," Bioinformatics **37**, 3734–3743 (2021).
16. Z. Xi, L. Liu, J. S. Rest, and C. C. Davis, "Coalescent versus Concatenation Methods and the Placement of Amborella as Sister to Water Lilies," Syst. Biol. **63**, 919–932 (2014).
17. B. T. Drew, B. R. Ruhfel, S. A. Smith, M. J. Moore, B. G. Briggs, M. A. Gitzendanner, P. S. Soltis, and D. E. Soltis, "Another Look at the Root of the Angiosperms Reveals a Familiar Tale," Syst. Biol. **63**, 368–382 (2014).
18. V. V. Goremykin, S. V. Nikiforova, P. J. Biggs, B. Zhong, P. Delange, W. Martin, S. Woetzel, R. A. Atherton, P. A. Mclenachan, and P. J. Lockhart, "The Evolutionary Root of Flowering Plants," Syst. Biol. **62**, 50–61 (2012).

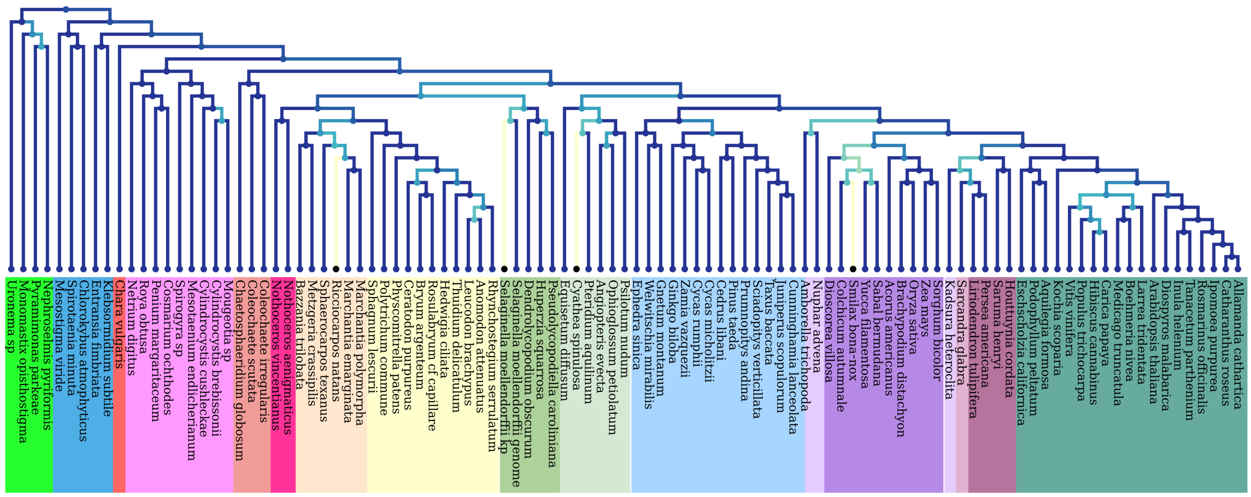

(a) QFM-FI

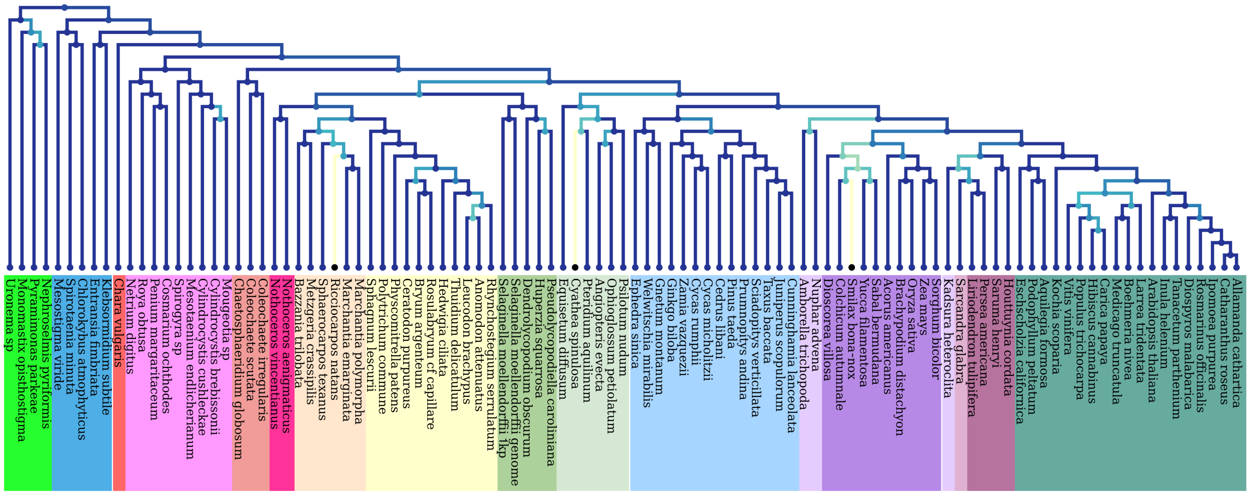

(b) QFM-PAUP

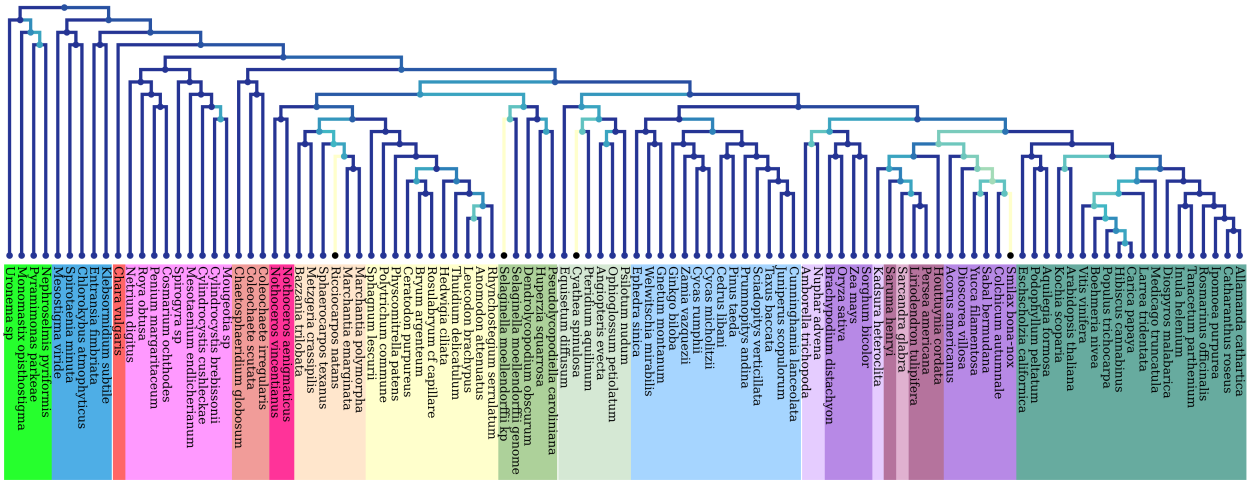

(c) QMC

Fig. S4. Study of the plant dataset using QFM-FI, QFM-PAUP and QMC. Here, sequences of 424 gene trees are used as input and svdQuartets method is used to generate unweighted quartets. The yellow to blue color scheme, as rendered by Phylo.io, indicates the similarity of best matching subtrees, with reference to the ASTRAL tree of Figure 3 of the main manuscript.

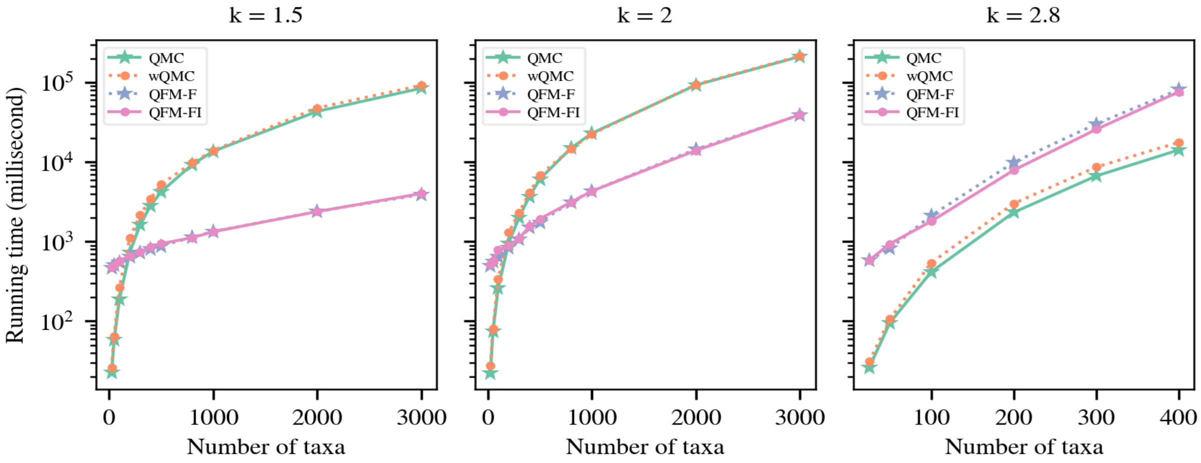

(a) Consistency level = 70%

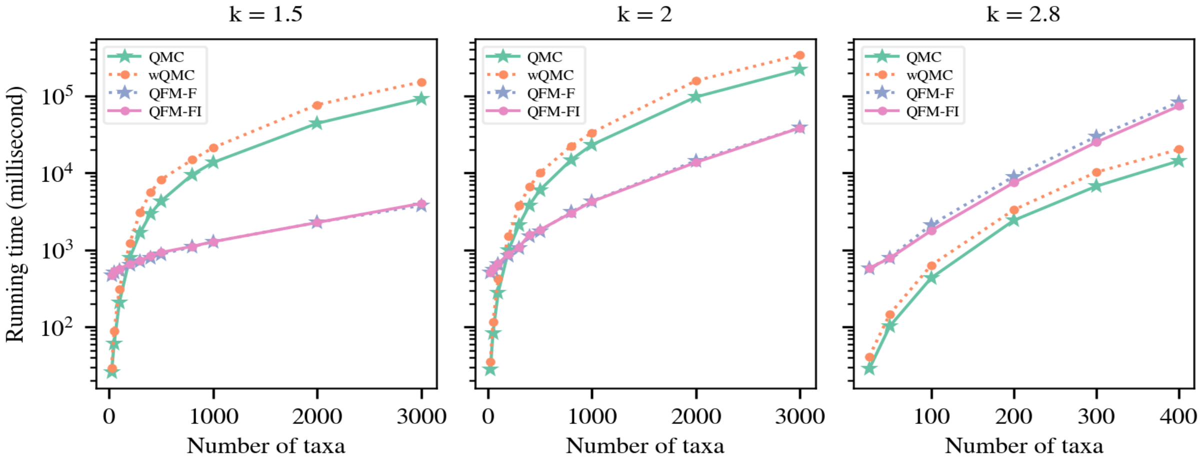

(b) Consistency level = 80%

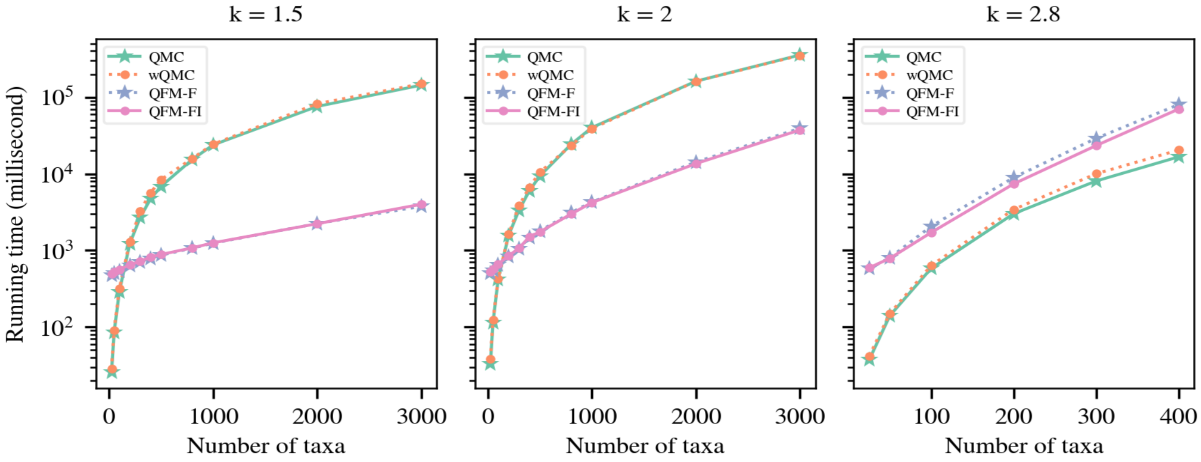

(c) Consistency level = 90%

Fig. S5. Running time comparison of QFM-F, QFM-FI, QMC and wQMC in logarithmic scale.

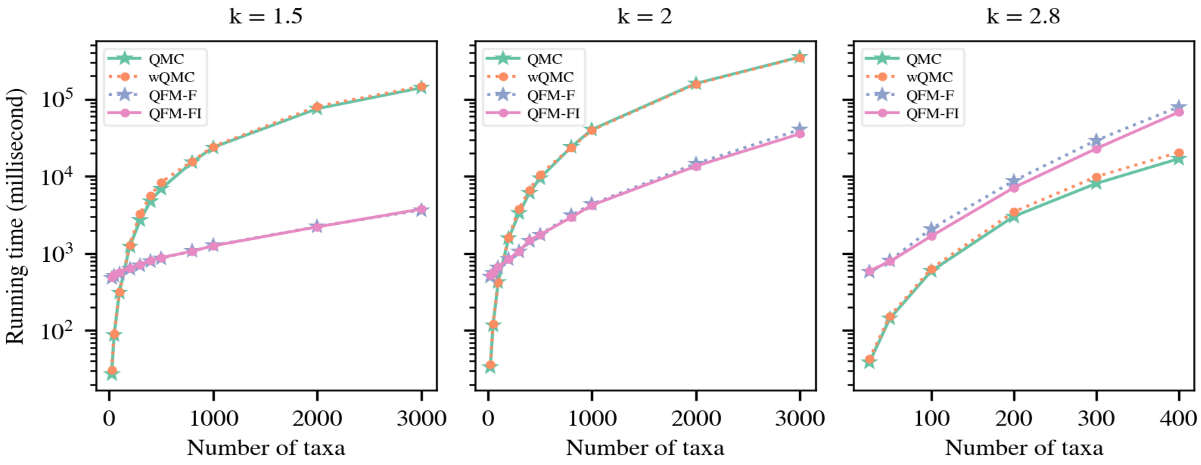

(d) Consistency level = 95%

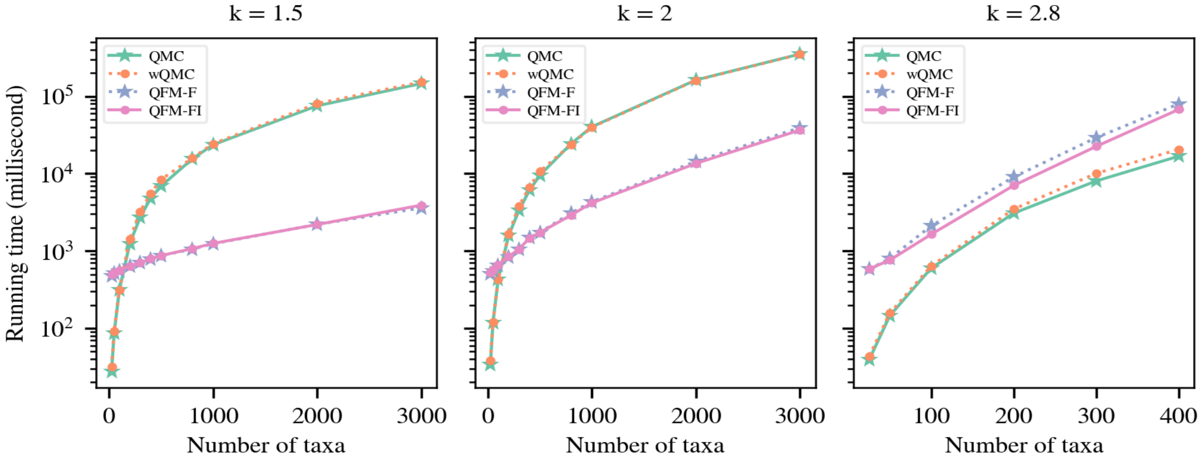

(e) Consistency level = 100%

Fig. S5. Running time comparison of QFM-F, QFM-FI, QMC and wQMC in logarithmic scale.

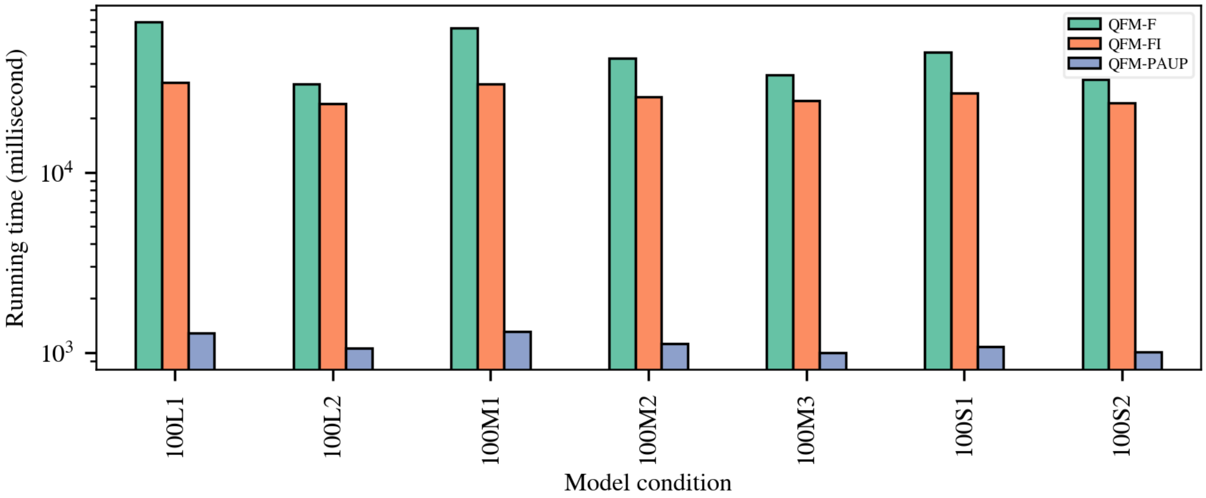

(a) Number of taxa = 100

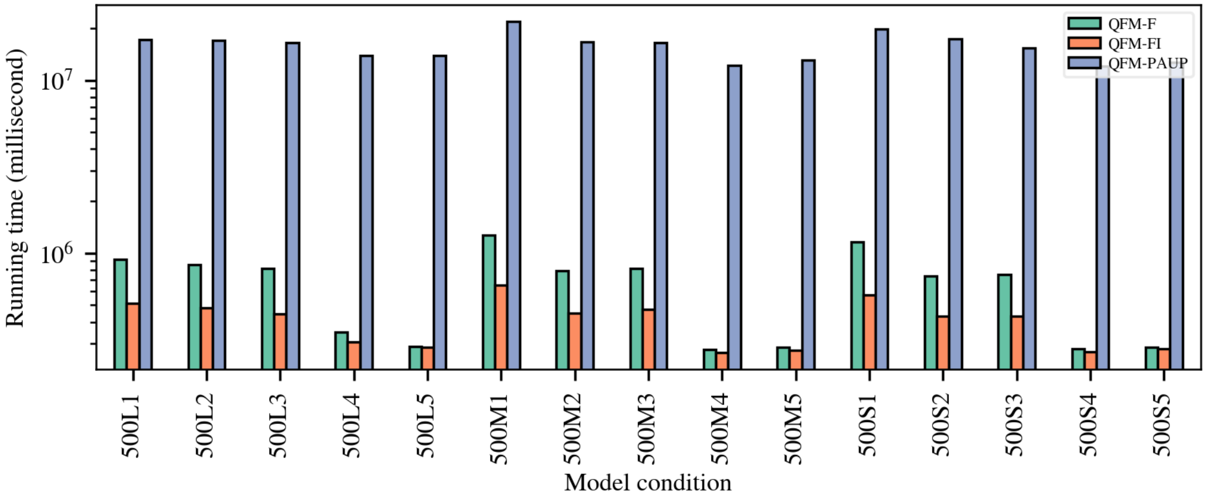

(b) Number of taxa = 500

Fig. S6. Running time comparison between QFM-F, QFM-FI and QFM-PAUP in logarithmic scale.

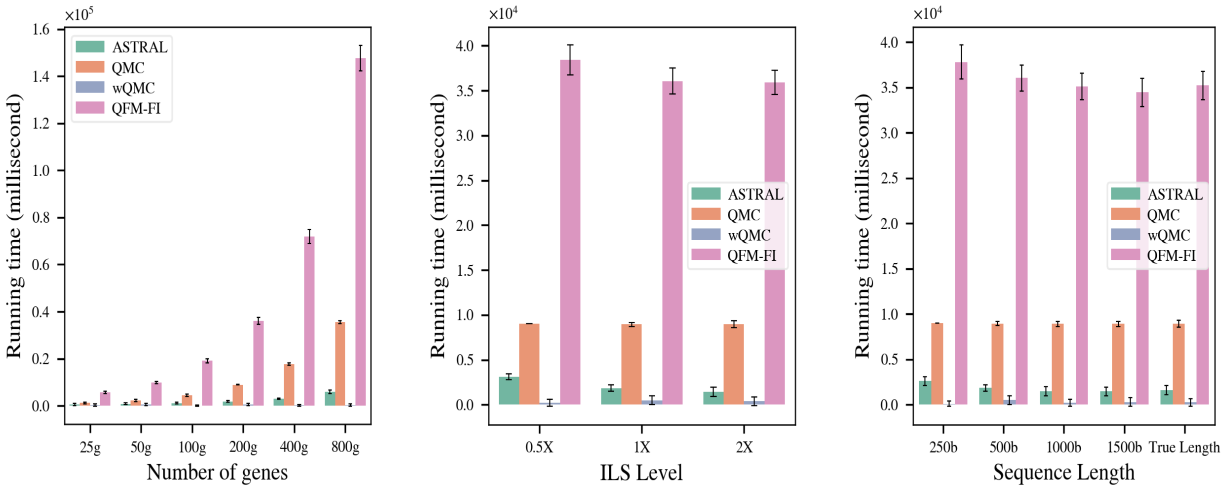

(a) Varying numbers of genes **(b)** Varying ILS level **(c)** Varying sequence length

Fig. S7. Running time comparison on 37-taxon simulated dataset in logarithmic scale.

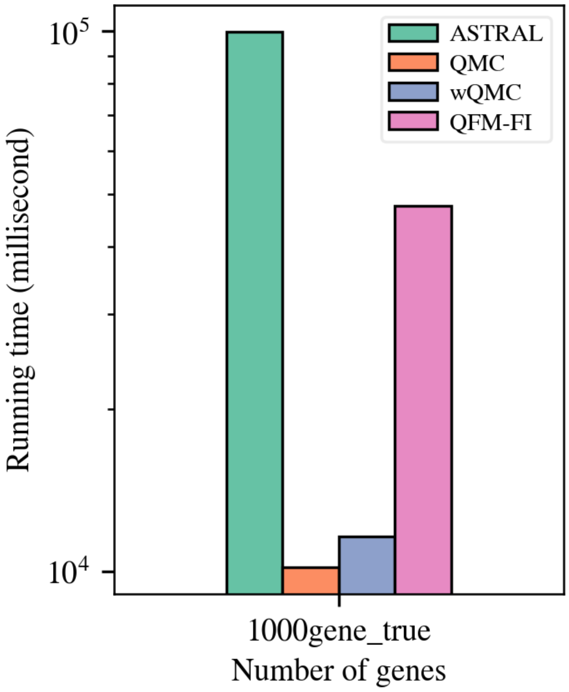

Fig. S8. Running time comparison on 100-taxon simulated dataset in logarithmic scale.

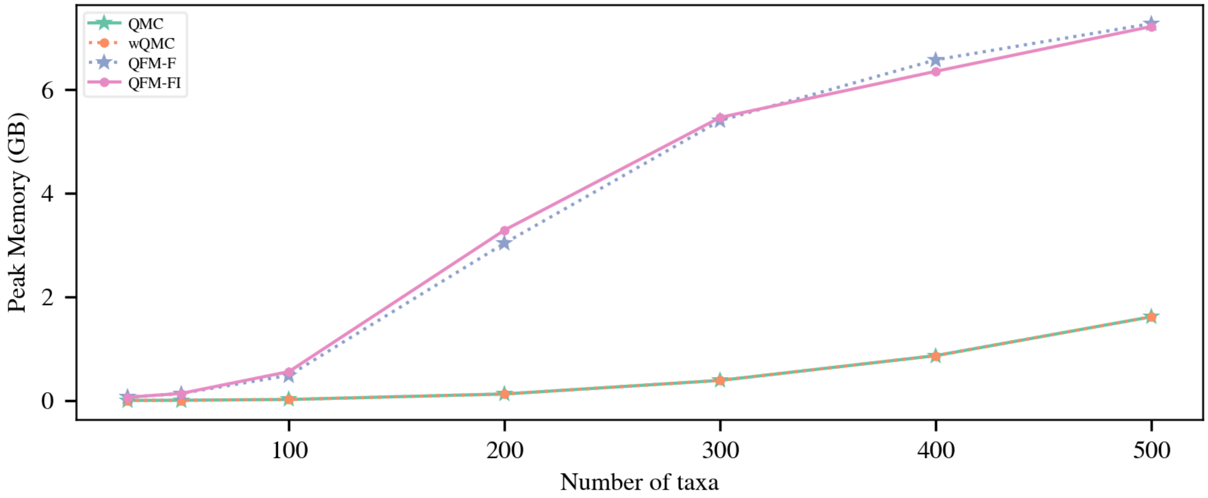

Fig. S9. Memory analysis of QMC, wQMC, QFM-F and QFM-FI for various number of taxa on simulated dataset 1. Here consistency is 70% and k= 2.8.

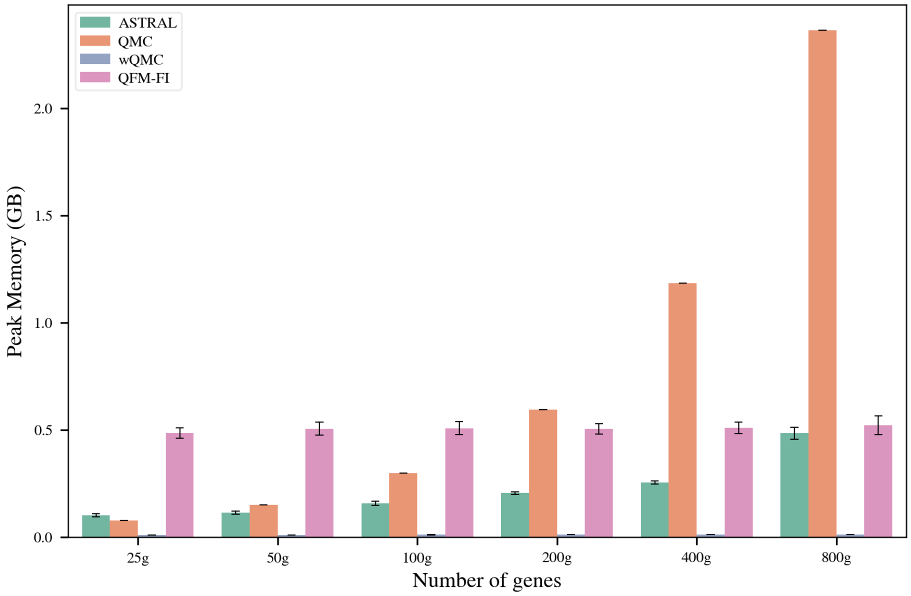

Fig. S10. Memory analysis on 37-taxon simulated dataset using QFM-FI, QMC, wQMC and ASTRAL by varying number of genes. ILS level is moderate and sequence length is 500bp.

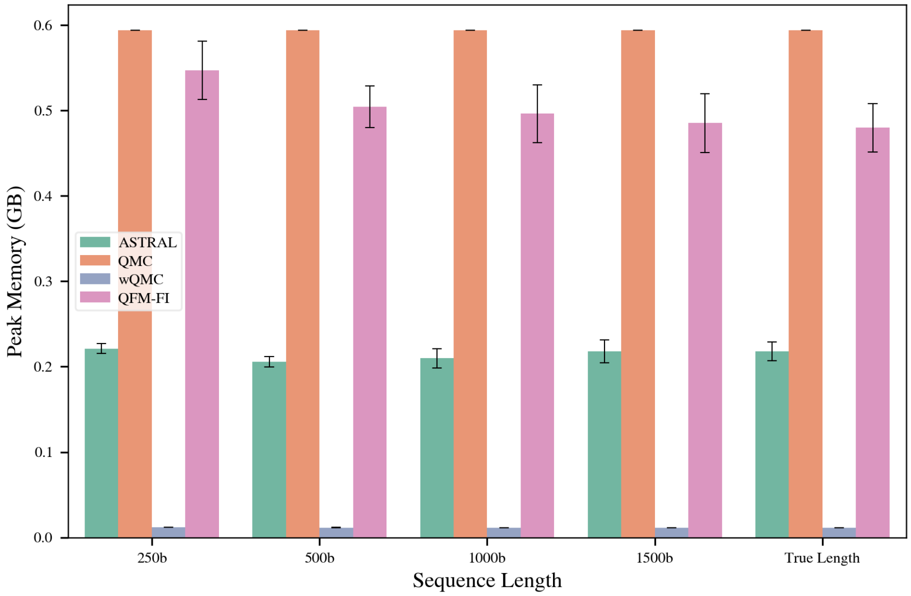

Fig. S11. Memory analysis on 37-taxon simulated dataset using QFM-FI, QMC, wQMC and ASTRAL by varying sequence length. The ILS level is moderate and the number of genes is 200.

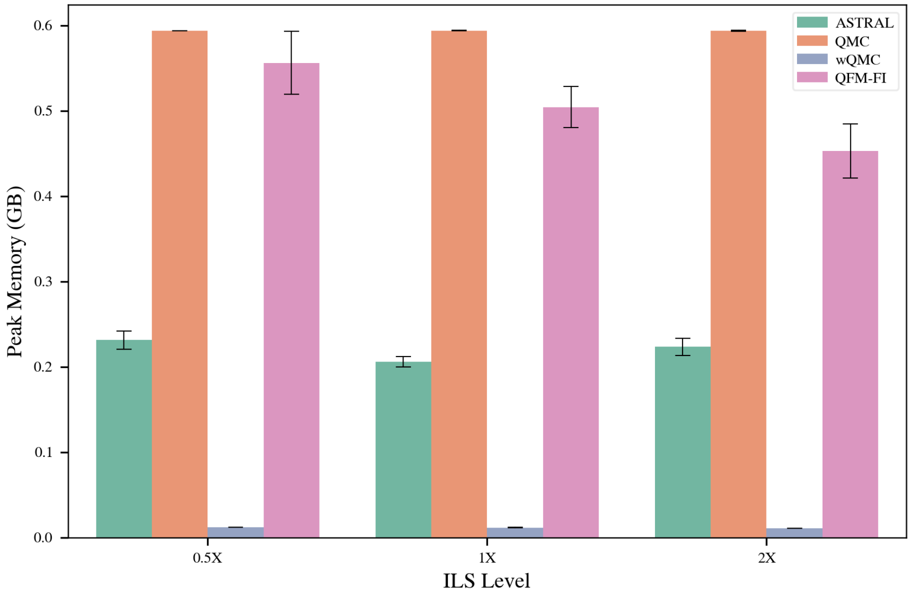

Fig. S12. Memory analysis on 37-taxon simulated dataset using QFM-FI, QMC, wQMC and ASTRAL by ILS level. Here, the number of genes is 200 and sequence length is 500bp.

Table S4. Statistical significance of the differences between QFM-F and QFM-FI on different model conditions. The p-values indicating statistically significant differences (i.e., $p \leq 0.05$) are shown in bold.

n	*k*	*p-value*				
		Correct = 70%	*Correct = 80%*	*Correct = 90%*	*Correct = 95%*	*Correct = 100%*
25	1.5	8.24E-01	0	3.02E-03	**0**	7.34E-01
25	2	**4.93E-02**	**1.08E-03**	**1.12E-03**	**8.32E-03**	8.33E-02
25	2.8	**2.90E-04**	**1.43E-02**	0	0	0
50	1.5	4.59E-02	9.70E-01	4.67E-01	4.91E-01	2.06E-01
50	2	**2.33E-04**	2.28E-01	2.80E-01	4.11E-01	9.44E-01
50	2.8	**9.03E-05**	**9.75E-05**	**2.99E-03**	**1.43E-02**	0
100	1.5	4.20E-01	6.52E-01	**4.34E-03**	6.23E-01	1.52E-01
100	2	**3.81E-03**	3.13E-01	7.37E-01	2.80E-01	5.19E-01
100	2.8	**1.91E-06**	**1.91E-06**	**1.22E-04**	**1.93E-03**	0
200	1.5	4.45E-01	**2.61E-01**	**2.11E-01**	**6.78E-01**	**6.70E-01**
200	2	**4.83E-04**	**2.39E-02**	**2.11E-01**	**3.99E-02**	8.08E-01
200	2.8	**1.91E-06**	**1.91E-06**	**9.04E-05**	**1.27E-04**	0
300	1.5	5.85E-01	1.90E-01	**3.79E-01**	**4.10E-01**	**7.68E-01**
300	2	**7.30E-03**	**1.60E-02**	8.12E-01	7.08E-01	**1.30E-01**
300	2.8	**1.91E-06**	**1.91E-06**	**1.91E-06**	**1.91E-06**	0
400	1.5	**6.37E-01**	6.48E-01	**7.36E-01**	**9.40E-01**	**6.46E-01**
400	2	**7.08E-04**	**4.30E-01**	**6.48E-01**	**4.98E-01**	2.40E-01
400	2.8	**1.91E-06**	**1.91E-06**	**1.91E-06**	**1.91E-06**	0
500	1.5	7.15E-02	**4.98E-01**	**8.98E-01**	**7.23E-01**	**6.74E-01**
500	2	**2.66E-02**	**6.29E-05**	**2.25E-01**	**9.73E-02**	**1.23E-01**
500	2.8	**1.91E-06**	**1.91E-06**	**1.91E-06**	**1.91E-06**	0
800	1.5	**1.35E-02**	**3.88E-01**	**3.82E-02**	**5.43E-02**	**8.37E-01**
800	2	**8.20E-05**	**3.65E-03**	**1.89E-01**	**2.40E-01**	**7.94E-01**
800	2.8	**1.91E-06**	**1.91E-06**	**1.91E-06**	**1.91E-06**	0
1000	1.5	**8.31E-03**	**1.77E-02**	**3.22E-01**	**9.26E-01**	**4.65E-01**
1000	2	**1.14E-01**	**4.86E-03**	**8.48E-03**	**7.29E-01**	**8.41E-01**
2000	1.5	**8.31E-03**	**1.26E-01**	**1.89E-01**	**9.85E-01**	**4.75E-01**
2000	2	**1.91E-05**	**1.89E-01**	**2.40E-02**	**2.77E-01**	**4.58E-02**
3000	1.5	**1.77E-01**	**5.53E-04**	**4.90E-01**	**8.37E-01**	**2.95E-01**
3000	2	**1.91E-06**	**2.67E-05**	**1.23E-01**	**5.46E-01**	**1.43E-01**

Table S5. Statistical significance of the differences between QFM-F and QMC on different model conditions. The p-values indicating statistically significant differences (i.e., $p \leq 0.05$) are shown in bold.

n	k	p-value				
		Correct = 70%	*Correct = 80%*	*Correct = 90%*	*Correct = 95%*	*Correct = 100%*
25	1.5	2.49E-01	3.88E-01	1.23E-01	**1.92E-02**	2.33E-03
25	2	6.25E-01	2.31E-01	5.04E-03	1.80E-03	2.83E-01
25	2.8	2.90E-04	1.43E-02	0	0	0
50	1.5	9.85E-01	2.94E-01	2.94E-01	2.45E-01	7.56E-01
50	2	9.46E-03	4.67E-01	6.22E-01	8.98E-01	5.38E-01
50	2.8	1.91E-05	9.37E-05	2.99E-03	1.43E-02	0
100	1.5	1.57E-01	8.69E-01	7.01E-01	7.29E-01	5.32E-02
100	2	3.62E-02	6.96E-02	5.71E-01	8.98E-01	6.48E-01
100	2.8	1.91E-06	1.91E-06	1.18E-04	7.05E-04	0
200	1.5	6.23E-01	1.14E-01	**7.30E-03**	**1.34E-05**	**3.95E-04**
200	2	1.65E-01	6.39E-03	2.45E-01	9.56E-01	7.29E-01
200	2.8	1.91E-06	1.91E-06	9.11E-05	1.27E-04	0
300	1.5	1.69E-02	2.02E-01	**1.91E-06**	**1.34E-05**	**1.91E-06**
300	2	4.00E-02	6.74E-01	3.68E-01	1.14E-01	**2.96E-02**
300	2.8	1.91E-06	1.91E-06	1.91E-06	9.03E-05	0
400	1.5	**4.73E-02**	9.73E-02	**1.07E-02**	**1.91E-05**	**1.68E-04**
400	2	4.75E-01	**8.31E-03**	**2.40E-02**	1.77E-01	1.77E-01
400	2.8	1.91E-06	1.91E-06	1.91E-06	1.91E-06	0
500	1.5	7.29E-01	**1.43E-03**	**3.81E-06**	**2.67E-05**	**3.81E-06**
500	2	4.75E-01	**4.41E-02**	**8.51E-04**	**1.36E-02**	**4.84E-02**
500	2.8	1.91E-06	1.91E-06	1.91E-06	1.91E-06	0
800	1.5	1.00E+00	**3.81E-06**	**1.91E-06**	**1.91E-06**	**1.91E-06**
800	2	**1.99E-03**	**5.72E-06**	**1.91E-06**	**1.91E-06**	**3.81E-06**
800	2.8	1.91E-06	1.91E-06	1.91E-06	1.91E-06	0
1000	1.5	5.63E-01	**1.91E-06**	**3.81E-06**	**1.91E-06**	**1.91E-06**
1000	2	**5.58E-03**	**2.61E-04**	**1.91E-06**	**3.81E-06**	**3.81E-06**
2000	1.5	7.59E-02	**1.91E-06**	**1.91E-06**	**1.91E-06**	**1.91E-06**
2000	2	**1.91E-06**	**1.91E-06**	**1.91E-06**	**1.91E-06**	**1.91E-06**
3000	1.5	7.59E-02	**1.91E-06**	**1.91E-06**	**1.91E-06**	**1.91E-06**
3000	2	**3.81E-06**	**1.91E-06**	**1.91E-06**	**1.91E-06**	**1.91E-06**

Table S6. Statistical significance of the differences between QFM-F and wQMC on different model conditions. The p-values indicating statistically significant differences (i.e., $p \leq 0.05$) are shown in bold.

n	*k*	*p-value*				
		Correct = 70%	*Correct = 80%*	*Correct = 90%*	*Correct = 95%*	*Correct = 100%*
25	1.5	4.18E-01	**1.21E-02**	3.88E-01	**5.22E-01**	1.72E-02
25	2	**1.31E-04**	5.22E-01	7.91E-02	7.92E-01	4.10E-01
25	2.8	3.00E-04	1.43E-02	0	0	0
50	1.5	8.66E-01	9.73E-02	2.02E-01	9.85E-01	8.12E-01
50	2	9.56E-01	8.41E-01	5.38E-01	3.88E-01	1.77E-01
50	2.8	2.09E-04	8.97E-05	2.99E-03	1.43E-02	0
100	1.5	1.33E-01	9.56E-01	1.05E-01	1.33E-01	7.56E-01
100	2	3.12E-01	9.85E-01	8.12E-01	3.68E-01	6.37E-02
100	2.8	1.91E-06	1.91E-06	9.44E-05	2.04E-03	3.17E-01
200	1.5	1.29E-02	1.89E-01	**2.10E-04**	**3.81E-06**	**7.30E-03**
200	2	3.49E-01	**7.30E-03**	4.09E-01	4.75E-01	2.10E-04
200	2.8	1.91E-06	1.91E-06	1.33E-03	6.57E-04	3.17E-01
300	1.5	1.61E-01	**1.69E-03**	**1.91E-06**	**1.91E-06**	**1.91E-06**
300	2	4.30E-01	9.56E-01	**4.00E-02**	3.88E-01	**1.43E-01**
300	2.8	1.91E-06	1.91E-06	7.19E-04	6.31E-02	**1.91E-06**
400	1.5	**9.26E-01**	2.45E-01	**4.09E-01**	**1.92E-02**	**4.84E-02**
400	2	2.61E-01	**2.66E-02**	**1.36E-02**	5.71E-01	6.96E-02
400	2.8	1.91E-06	1.91E-06	9.25E-05	6.27E-04	**1.91E-06**
500	1.5	4.09E-01	**7.08E-04**	**1.99E-03**	**4.83E-04**	**3.81E-06**
500	2	8.97E-02	**1.02E-03**	**1.34E-04**	**4.98E-01**	**3.12E-01**
500	2.8	1.91E-06	1.91E-06	1.91E-06	1.61E-04	**1.29E-04**
800	1.5	**5.86E-04**	**1.91E-06**	**3.81E-06**	**1.91E-06**	**1.91E-06**
800	2	**6.48E-01**	**1.34E-05**	**5.72E-06**	**1.91E-05**	**6.74E-01**
800	2.8	1.91E-06	1.91E-06	1.91E-06	1.91E-06	**8.50E-05**
1000	1.5	**7.27E-04**	**1.91E-06**	**1.91E-06**	**1.91E-06**	**1.91E-06**
1000	2	**2.61E-04**	**1.91E-05**	**1.91E-05**	**2.67E-05**	**8.98E-01**
2000	1.5	**4.77E-05**	**1.91E-06**	**1.91E-06**	**1.91E-06**	**1.91E-06**
2000	2	**1.68E-04**	**1.91E-06**	**1.91E-06**	**1.91E-06**	**3.81E-06**
3000	1.5	**1.91E-06**	**1.91E-06**	**1.91E-06**	**1.91E-06**	**1.91E-06**
3000	2	**3.81E-06**	**1.91E-06**	**1.91E-06**	**1.91E-06**	**1.91E-06**

Table S7. Statistical significance of the differences between QFM-FI and QMC on different model conditions. The p-values indicating statistically significant differences (i.e., $p \leq 0.05$) are shown in bold.

n	*k*	*p-value*				
		Correct = 70%	*Correct = 80%*	*Correct = 90%*	*Correct = 95%*	*Correct = 100%*
25	1.5	2.04E-01	3.88E-01	3.88E-01	**1.92E-02**	8.31E-03
25	2	1.54E-01	1.00E+00	3.23E-01	8.33E-02	9.53E-02
25	2.8	0	0	0	0	0
50	1.5	5.69E-01	4.98E-01	4.09E-01	1.33E-01	4.98E-01
50	2	8.08E-01	7.51E-01	1.00E+00	1.00E+00	7.51E-01
50	2.8	3.17E-01	3.17E-01	0	0	0
100	1.5	1.46E-02	8.69E-01	2.31E-01	8.98E-01	1.14E-01
100	2	2.77E-01	8.97E-02	5.71E-01	2.31E-01	8.12E-01
100	2.8	3.89E-01	1.00E+00	3.17E-01	3.17E-01	0
200	1.5	6.80E-01	**4.41E-02**	**2.33E-03**	**2.67E-05**	**4.77E-05**
200	2	5.46E-01	2.94E-01	**4.00E-02**	2.94E-01	7.01E-01
200	2.8	2.78E-01	3.05E-01	3.17E-01	0	0
300	1.5	1.35E-02	4.98E-01	**3.81E-06**	**2.67E-05**	**1.91E-06**
300	2	9.85E-01	2.45E-01	5.96E-01	8.26E-02	1.23E-01
300	2.8	1.38E-01	4.83E-01	5.44E-01	5.85E-01	0
400	1.5	7.86E-02	1.23E-01	**1.21E-02**	**1.05E-04**	**8.20E-05**
400	2	**1.72E-02**	**1.72E-02**	**4.84E-02**	**3.28E-02**	2.77E-01
400	2.8	9.55E-01	1.94E-01	1.00E+00	3.17E-01	0
500	1.5	2.16E-01	**6.29E-05**	**9.54E-06**	**3.62E-05**	**3.81E-06**
500	2	8.98E-01	**2.61E-04**	**1.68E-04**	**1.21E-03**	1.33E-01
500	2.8	6.05E-01	8.64E-01	3.05E-01	3.17E-01	0
800	1.5	**3.62E-02**	**9.54E-06**	**1.91E-06**	**1.91E-06**	**1.91E-06**
800	2	**3.81E-06**	**1.91E-06**	**3.81E-06**	**5.72E-06**	**1.91E-06**
800	2.8	7.36E-01	4.97E-01	3.82E-01	9.32E-01	0
1000	1.5	**1.36E-02**	**1.91E-06**	**1.91E-06**	**1.91E-06**	**1.91E-06**
1000	2	**2.61E-04**	**9.54E-06**	**1.91E-06**	**5.72E-06**	**3.81E-06**
2000	1.5	**8.51E-04**	**1.91E-06**	**1.91E-06**	**1.91E-06**	**1.91E-06**
2000	2	**1.91E-06**	**1.91E-06**	**1.91E-06**	**1.91E-06**	**1.91E-06**
3000	1.5	**1.99E-03**	**1.91E-06**	**1.91E-06**	**1.91E-06**	**1.91E-06**
3000	2	**1.91E-06**	**1.91E-06**	**1.91E-06**	**1.91E-06**	**1.91E-06**

Table S8. Statistical significance of the differences between QFM-FI and wQMC on different model conditions. The p-values indicating statistically significant differences (i.e., $p \leq 0.05$) are shown in bold.

n	*k*	*p-value*				
		Correct = 70%	*Correct = 80%*	*Correct = 90%*	*Correct = 95%*	*Correct = 100%*
25	1.5	1.57E-01	**1.21E-02**	9.27E-01	**5.22E-01**	4.75E-01
25	2	**2.67E-05**	**5.83E-02**	**4.31E-01**	**7.69E-02**	9.12E-01
25	2.8	**8.33E-02**	**0**	0	0	0
50	1.5	6.80E-01	1.05E-01	1.89E-01	8.98E-01	5.71E-01
50	2	**1.07E-02**	6.48E-01	2.45E-01	1.89E-01	1.05E-01
50	2.8	**5.65E-02**	**1.00E+00**	**0**	**0**	0
100	1.5	3.32E-02	8.12E-01	**2.96E-02**	1.23E-01	4.52E-01
100	2	**3.62E-02**	4.75E-01	7.84E-01	7.84E-01	8.26E-02
100	2.8	**1.10E-01**	**3.05E-01**	**5.64E-01**	**5.64E-01**	3.17E-01
200	1.5	4.24E-02	**6.96E-02**	**2.61E-04**	**3.81E-06**	**7.30E-03**
200	2	**4.41E-02**	**3.15E-03**	**2.45E-01**	**8.98E-01**	7.08E-04
200	2.8	**4.70E-04**	**1.91E-06**	**1.91E-06**	**2.89E-03**	3.17E-01
300	1.5	1.37E-01	**4.86E-03**	**1.91E-06**	**1.91E-06**	**1.91E-06**
300	2	**1.92E-02**	**2.45E-01**	**2.96E-02**	4.75E-01	**6.39E-03**
300	2.8	**3.26E-03**	**1.63E-03**	**1.91E-06**	**1.91E-06**	**1.91E-06**
400	1.5	**7.23E-01**	2.45E-01	**4.98E-01**	**1.72E-02**	**4.00E-02**
400	2	**8.31E-03**	**6.39E-03**	**2.66E-02**	**2.45E-01**	5.32E-02
400	2.8	**1.70E-03**	**2.55E-03**	**1.91E-06**	**1.91E-06**	**1.91E-06**
500	1.5	**4.00E-02**	**2.67E-05**	**4.83E-04**	**5.86E-04**	**1.91E-05**
500	2	**1.07E-02**	**1.91E-05**	**4.77E-05**	**1.05E-01**	**1.54E-01**
500	2.8	**1.91E-06**	**1.91E-06**	**1.91E-06**	**1.91E-06**	**1.29E-04**
800	1.5	**1.05E-04**	**3.81E-06**	**1.91E-06**	**1.91E-06**	**1.91E-06**
800	2	**9.44E-03**	**1.91E-06**	**9.54E-06**	**1.91E-05**	**8.69E-01**
800	2.8	**1.91E-06**	**1.10E-04**	**1.91E-06**	**1.19E-04**	**8.50E-05**
1000	1.5	**2.67E-05**	**1.91E-06**	**1.91E-06**	**1.91E-06**	**1.91E-06**
1000	2	**1.91E-05**	**9.54E-06**	**9.54E-06**	**2.61E-04**	**8.41E-01**
2000	1.5	**1.91E-06**	**1.91E-06**	**1.91E-06**	**1.91E-06**	**1.91E-06**
2000	2	**1.34E-05**	**1.91E-06**	**1.91E-06**	**1.91E-06**	**1.91E-06**
3000	1.5	**1.91E-06**	**1.91E-06**	**1.91E-06**	**1.91E-06**	**1.91E-06**
3000	2	**1.91E-06**	**1.91E-06**	**1.91E-06**	**1.91E-06**	**1.91E-06**

Table S9. Statistical significance of the differences between QFM-FI and QFM_PAUP on different model conditions. The p-values indicating statistically significant differences (i.e., $p \leq 0.05$) are shown in bold.

Model condition	*No. of quartets*	*P-value*
100L1	3921225	.5616
100L2	3921225	.5641
100M1	3921225	.8425
100M2	3921225	.2926
100M3	3921225	.3173
100S1	3921225	.8345
100S2	3921225	.0835
500L1	36067497	**.0247**
500L2	36067497	.4478
500L3	36067497	.0700
500L4	36067497	.0716
500L5	36067497	.3217
500M1	36067497	.5246
500M2	36067497	.4992
500M3	36067497	.0669
500M4	36067497	**.0452**
500M5	36067497	.3686
500S1	36067497	.4683
500S2	36067497	.8906
500S3	36067497	.4648
500S4	36067497	.5010
500S5	36067497	.1660

Table S10. Statistical significance of the differences between QFM-FI and ASTRAL on different model conditions in 37-taxon simulated dataset. The p-values indicating statistically significant differences (i.e., $p \leq 0.05$) are shown in bold.

Model Condition			*p-value*
No. of genes	*Sequence length*	*ILS level*	
25 g	500 bp	Moderate (1X)	0.6178
50 g	500 bp	Moderate (1X)	0.6918
100 g	500 bp	Moderate (1X)	0.5654
200 g	250 bp	Moderate (1X)	**0.0273**
200 g	500 bp	High (0.5X)	0.4785
200 g	500 bp	Moderate (1X)	**0.0457**
200 g	500 bp	Low (2X)	0.3311
200 g	1000 bp	Moderate (1X)	0.0835
200 g	1500 bp	Moderate (1X)	0.0833
200 g	True length	Moderate (1X)	0.1573
400 g	500 bp	Moderate (1X)	0.3587
800 g	500 bp	Moderate (1X)	0.1573

Table S11. Statistical significance of the differences between QFM-FI and QMC on different model conditions in 37-taxon simulated dataset. The p-values indicating statistically significant differences (i.e., $p \leq 0.05$) are shown in bold.

Model Condition			*p-value*
No. of genes	*Sequence length*	*ILS level*	
25 g	500 bp	Moderate (1X)	0.8824
50 g	500 bp	Moderate (1X)	0.2987
100 g	500 bp	Moderate (1X)	0.8344
200 g	250 bp	Moderate (1X)	**0.0061**
200 g	500 bp	High (0.5X)	0.1728
200 g	500 bp	Moderate (1X)	**0.0457**
200 g	500 bp	Low (2X)	0.3311
200 g	1000 bp	Moderate (1X)	0.0835
200 g	1500 bp	Moderate (1X)	**0.0458**
200 g	True length	Moderate (1X)	0.0833
400 g	500 bp	Moderate (1X)	0.3587
800 g	500 bp	Moderate (1X)	0.1573

Table S12. Statistical significance of the differences between QFM-FI and wQMC on different model conditions in 37-taxon simulated dataset. The p-values indicating statistically significant differences (i.e., $p \leq 0.05$) are shown in bold.

Model Condition			***p-value***
No. of genes	***Sequence length***	***ILS level***	
25 g	500 bp	Moderate (1X)	0.6051
50 g	500 bp	Moderate (1X)	0.7072
100 g	500 bp	Moderate (1X)	0.5654
200 g	250 bp	Moderate (1X)	**0.0061**
200 g	500 bp	High (0.5X)	0.1728
200 g	500 bp	Moderate (1X)	**0.0457**
200 g	500 bp	Low (2X)	0.3311
200 g	1000 bp	Moderate (1X)	0.0835
200 g	1500 bp	Moderate (1X)	**0.0458**
200 g	True length	Moderate (1X)	0.0833
400 g	500 bp	Moderate (1X)	0.3587
800 g	500 bp	Moderate (1X)	0.1573

Table S13. Quartet scores on biological datasets.

Dataset	***Method***	***Number of input quartets***	***Quartet score***	***Normalized quartet score (%)***
Angiosperm	QFM-FI	14499592	11553053	79.68
	QMC		11551948	79.67
	wQMC		11551948	79.67
	ASTRAL		11553053	79.68
Amniota	QFM-FI	125412	83604	66.66
	QMC		83604	66.66
	wQMC		83604	66.66
	ASTRAL		83604	66.66
Avian	QFM-FI	2462111516	1216535727	49.91
	QMC		Aborted	Aborted
	wQMC		1223578787	49.70
	ASTRAL		1231992828	50.04
Plant (1st experimental setting)	QFM-FI	378936182	339020685	89.47
	QMC		339021575	89.47
	wQMC		339021575	89.47
	ASTRAL		339023690	89.47
